# A novel zebrafish intestinal tumor model reveals a role for *cyp7a1*-dependent tumor–liver crosstalk in causing adverse effects on the host

**DOI:** 10.1242/dmm.032383

**Published:** 2018-05-03

**Authors:** Sora Enya, Koichi Kawakami, Yutaka Suzuki, Shinpei Kawaoka

**Affiliations:** 1 Advanced Telecommunications Research Institute International (ATR), The Thomas N. Sato BioMEC-X Laboratories, Kyoto 619-0288, Japan; 2 ERATO Sato Live Bio-forecasting Project, Japan Science and Technology Agency (JST), Kyoto 619-0288, Japan; 3 Division of Molecular and Developmental Biology, National Institute of Genetics, and Department of Genetics, SOKENDAI (The Graduate University for Advanced Studies), Mishima, Shizuoka 411-8540, Japan; 4 The University of Tokyo, Graduate School of Frontier Science, Kashiwa 277-8651, Japan

**Keywords:** Intestinal tumor, Hepatomegaly, Liver inflammation, Growth defect, *cyp7a1*, Cholesterol metabolism

## Abstract

The nature of host organs and genes that underlie tumor-induced physiological disruption on the host remains ill-defined. Here, we establish a novel zebrafish intestinal tumor model that is suitable for addressing this issue, and find that hepatic *cyp7a1*, the rate-limiting factor for synthesizing bile acids [or, in the case of zebrafish, bile alcohol (BA)], is such a host gene. Inducing *kras^G12D^* by *Gal4* specifically expressed in the posterior intestine resulted in the formation of an intestinal tumor. The local intestinal tumor caused systemic detrimental effects on the host, including liver inflammation, hepatomegaly, growth defects and organismal death. Whole-organism-level gene expression analysis and metabolite measurements revealed that the intestinal tumor reduced total BA levels, possibly via altered expression of hepatic *cyp7a1*. Genetically overexpressing *cyp7a1* in the liver restored BA synthesis and ameliorated tumor-induced liver inflammation, but not other tumor-dependent phenotypes. Thus, we found a previously unknown role of *cyp7a1* as the host gene that links the intestinal tumor, hepatic cholesterol–BA metabolism and liver inflammation in tumor-bearing zebrafish larvae. Our model provides an important basis to discover host genes responsible for tumor-induced phenotypes and to uncover mechanisms underlying how tumors adversely affect host organisms.

## Abstract (precis)

**Summary:** The authors genetically dissect the adverse effects on the host in a new model of zebrafish intestinal tumor.

## INTRODUCTION

Tumors disrupt host physiology in various ways, ultimately leading to organismal death (Egeblad et al., 2010; Fearon et al., 2012; McAllister and Weinberg, 2014; Owusu-Ansah and Perrimon, 2015). Mechanisms underlying physiological disruption by tumors involve inter-organ communication between tumors and normal organs. Due to its complex nature, how tumors affect host organs, and when and how host organs detect and respond to tumors, have remained largely elusive. In particular, host genes and signaling cascades mediating tumor–organ interaction (and thus tumor-induced phenotypes) are poorly defined. Understanding the nature of tumor–organ interaction and its mediator(s) at the genetic level is essential to understand how tumors interfere with host physiology, and to suggest a therapy that buffers tumor-dependent physiological disruption on the host.

Animal models that are amenable to whole-organism-level experiments and genetic manipulations provide a tool for discovering physiologically important tumor–organ interaction and underlying mechanisms behind them. The fly *Drosophila melanogaster* is one such model. A fly tumor originating from the eye imaginal disc secretes insulin-like peptide 8 (Dilp8) to delay organismal growth and maturation, thereby enabling, or forcing, the organism to coordinate their overall growth with a local disease state (Garelli et al., 2012). Consistent with local disrupted states having influence on distant processes such as growth, physiological disruption such as wounding also induces a Dilp8-dependent growth delay (Colombani et al., 2015, 2012; Garelli et al., 2012, 2015; Katsuyama et al., 2015; Owusu-Ansah and Perrimon, 2015; Vallejo et al., 2015). In this phenomenon, Lgr3, the receptor for Dilp8 expressed in neurons, is the host protein responsible for the tumor-dependent growth delay (Colombani et al., 2015; Garelli et al., 2015; Vallejo et al., 2015). These studies establish the concept that organisms are able to sense local physiological disruption that can be spread systemically (Owusu-Ansah and Perrimon, 2015). Others have shown that fly tumors produce ImpL2, an antagonist for insulin-like growth factors, to cause loss of peripheral tissues, including muscle and fat: a phenomenon called cachexia (Fearon et al., 2012; Figueroa-Clarevega and Bilder, 2015; Kwon et al., 2015). Such hormone-mediated mechanisms of cancer-induced cachexia have also been reported in mice. For example, lung cancer secretes parathyroid-related hormone (PTHrP), which increases fat thermogenesis through its receptor, PTHR, encoded by a host gene that is expressed in fat cells, resulting in cachexia (Kir et al., 2016, 2014). In another example, adipose triglyceride lipases have been implicated in cachexia, since mice lacking these lipases become resistant to cancer-induced fat loss (Das et al., 2011). In addition, tumors often elicit massive inflammation in distant organs, which is thought to affect whole-organism physiology (Egeblad et al., 2010; Fearon et al., 2012; McAllister and Weinberg, 2014; Owusu-Ansah and Perrimon, 2015). These tumor-induced phenomena are highly heterogeneous: the same tumors do not always cause the same systemic phenotypes (Fearon et al., 2012). This indicates that these phenotypes are influenced by host genotype and physiology, and vice versa, and thus appear to behave in a context-dependent manner. Most importantly, as described above, even in cachexia, a well-known tumor-induced phenotype, only a small set of host genes responsible for this phenomenon have been identified.

Zebrafish is an emerging model for studying tumors (White et al., 2013) and tumor–organ interaction due to its plethora of advantages, including: (i) they are a vertebrate that gives rise to numerous offspring at once, (ii) larvae are transparent, enabling researchers to observe tumorigenesis and tumor-induced phenotypes easily in live animals, (iii) they are small enough to allow whole-organism-level experiments and (iv) genetic manipulations are relatively easy and affordable, especially when compared with mice. As a good example, zebrafish melanoma models have provided various insights into melanoma development *in vivo* (Kaufman et al., 2016; Lister et al., 2014; Santoriello et al., 2010; White et al., 2011). Zebrafish genetic tumor models that are currently available often develop tumors at relatively later stages of zebrafish development, mostly after pigmentation (White et al., 2013). In such cases, it takes time (several weeks to months) to obtain tumor-bearing fish, and they are already opaque when tumors arise unless the *casper* mutation is introduced (White et al., 2008). Hence, it would be meaningful to create a novel zebrafish tumor model where tumor formation and proliferation occur in the transparent stage of zebrafish development. Furthermore, as is the case for zebrafish, most animal tumor models develop tumors at an adult stage, thereby preventing investigation into how tumors affect growing, juvenile vertebrates. For these reasons, a novel zebrafish tumor model is required.

In the current study, we successfully generated a novel intestinal tumor model. Careful characterization of this model led to the identification of four tumor-induced phenotypes that are seen even in human cancer patients: systemic inflammation, hepatomegaly, growth defects and organismal death. Anomalies in gene expression and metabolism were found in both the intestinal tumor and the distant liver upon whole-organism transcriptome analysis. On the basis of these, we found that a tumor–liver crosstalk, which can be defined by expression of hepatic *cyp7a1* accompanied by altered cholesterol–bile alcohol (BA) flux, promote infiltration of neutrophils to the liver (liver inflammation) in tumor-bearing larvae.

## RESULTS

### *pInt-Gal4*-driven *kras^G12D^* expression causes outgrowth of posterior intestine, leading to formation of the intestinal tumor

**Fig. 1.**
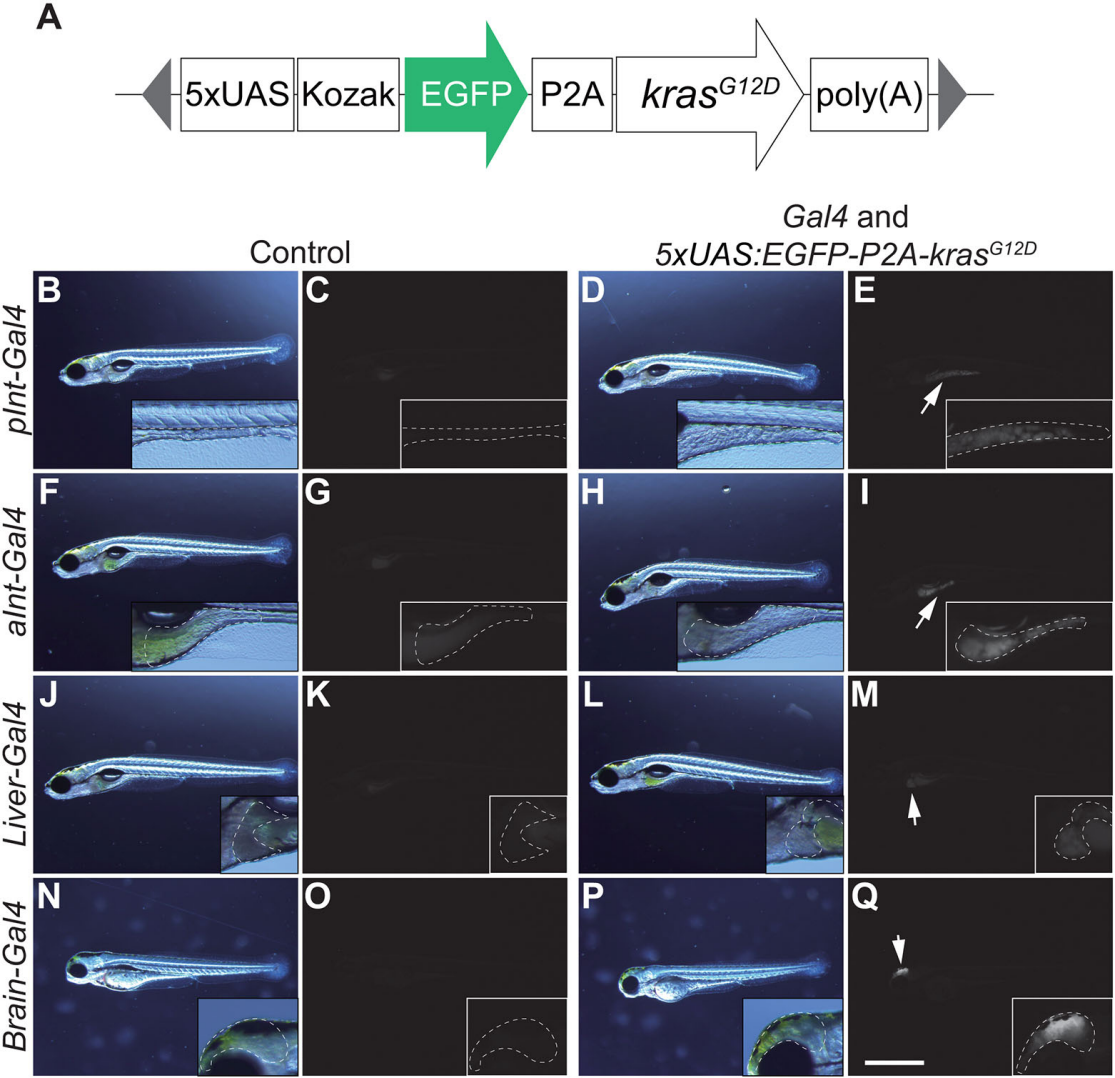
**Screening for *Gal4* lines that induce outgrowth of target organs when crossed with *Tg(5×UAS:EGFP-P2A-kras^G12D^)*.** (A) The structure of *5×UAS:EGFP-P2A-kras^G12D^*. The gray triangles represent the sequence recognized by Tol2 transposases. (B-Q) Screening for a *Gal4* line that is potent to induce outgrowth of target organs. Images of the sibling control (left) and *EGFP-kras^G12D^*-expressing larvae (right) are shown. Higher-magnification images are also presented. Images on the left for each group are bright-field images, whereas the others are fluorescence images (EGFP). Target organs are outlined by a dashed white line for (B-E) gSAIzGFFD1105A (*pInt-Gal4*) (7 dpf), (F-I) gSAIzGFFM103B (*aInt-Gal4*) (7 dpf), (J-M) gSAIzGFFD886A (*Liver-Gal4*) (7 dpf) and (N-Q) gSAGFF138A (*Brain-Gal4*) (3 dpf). Larvae without EGFP expression from the same clutch were used as sibling controls. White arrows indicate organs that express the *EGFP-kras^G12D^* transgene. Scale bar: 1 mm. Data are representative of at least two independent experiments.

In order to generate a zebrafish model of tumorigenesis with early onset, we sought for a *Gal4* line(s) capable of driving gene expression to a single organ (i.e. organ specificity) at an early stage of zebrafish development. To this end, we crossed a set of *Gal4* lines (Asakawa and Kawakami, 2008; Asakawa et al., 2008) with a line generated in this study [*Tg(5×UAS:EGFP-P2A-kras^G12D^)*] by using the *Tol2* system (Fig. 1A and Table S1) (Kawakami, 2004; Kawakami et al., 1998)*. Tg(5×UAS:EGFP-P2A-kras^G12D^)* harbored a mutated *kras* gene, *kras^G12D^*, one of the most prevalent driver oncogenes in human malignant tumors (Fig. 1A and Table S1) (Schubbert et al., 2007). Expression of *kras^G12D^* was linked with *EGFP* expression by porcine teschovirus-1 2A (*P2A*), a self-cleaving peptide sequence (Kim et al., 2011). Tissue outgrowth of *kras^G12D^*-positive cells was examined using a fluorescence stereoscopic microscope within approximately 48 h after observation of *Gal4*-dependent EGFP expression in a target organ.

Lines were identified that showed the requisite expression in posterior intestinal cells (gSAIzGFFD1105A; *pInt-Gal4*), anterior intestinal cells (gSAIzGFFM103B; *aInt-Gal4*), brain (gSAGFF138A; *Brain-Gal4*) and liver (gSAIzGFFD886A; *Liver-Gal4*) (Fig. 1). From these, *pInt-Gal4* was chosen for further characterization due to its ability to cause efficient outgrowth of posterior intestinal cells upon *kras^G12D^* expression (Fig. 1B-E). *aInt-Gal4* was also able to cause outgrowth of anterior intestinal cells (Fig. 1F-I). However, outgrowth of intestinal cells by *aInt-Gal4* was less dramatic when compared to that by *pInt-Gal4*. Moreover, expression of *aInt-Gal4*, despite being specific after 5 dpf, was somewhat non-specific during 2-4 dpf, leading to abnormal growth of epidermal cells in a temporal manner (Fig. S1A-D).

**Fig. 2.**
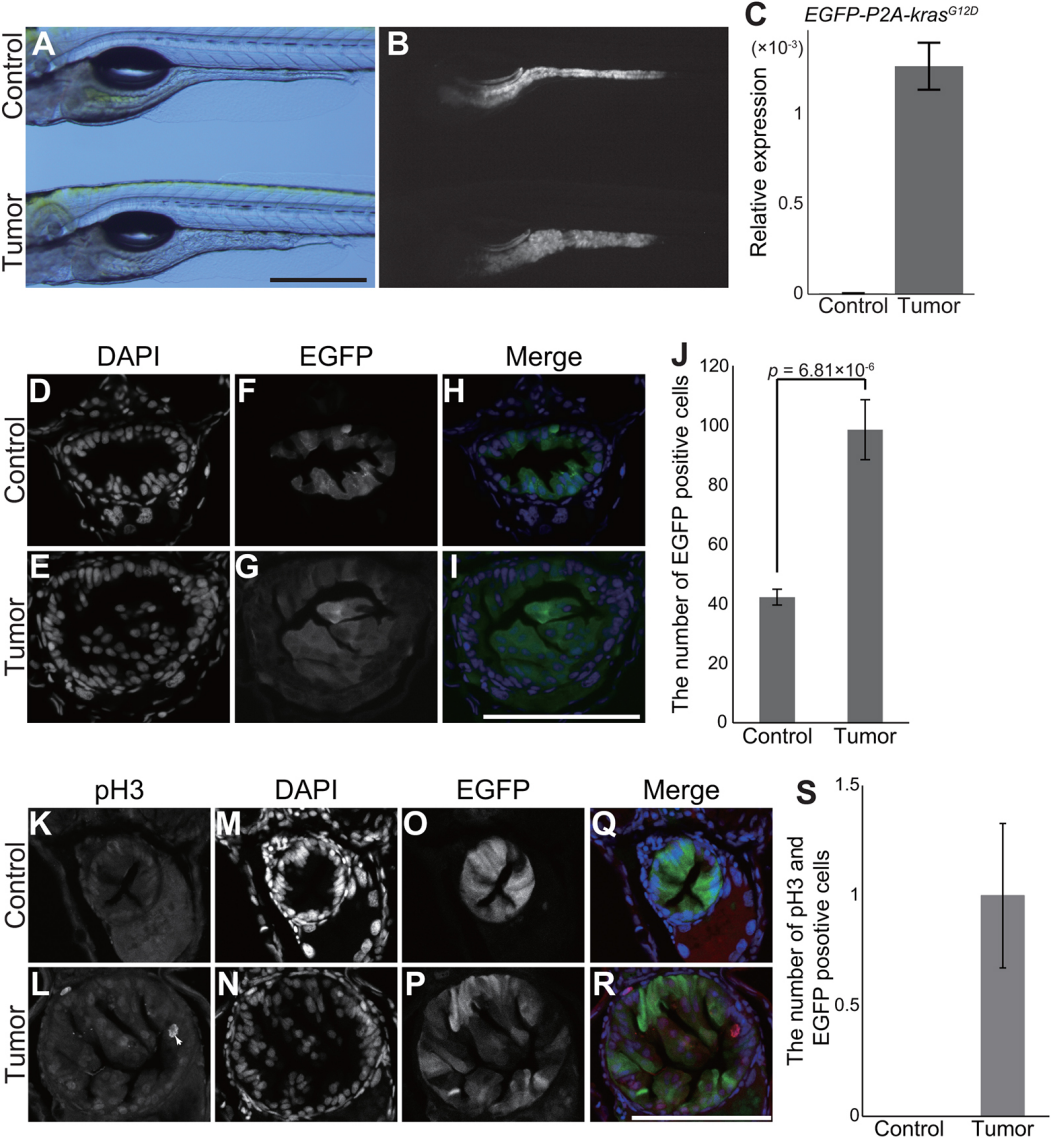
***pInt-Gal4*-driven *kras^G12D^* expression leads to abnormal proliferation of intestinal cells.** (A,B) Representative images of tumor-bearing larvae [*Tg(pInt-Gal4)^+/Tg^; Tg(5×UAS:EGFP-P2A-kras^G12D^)^+/Tg^*] and the sibling controls [*Tg(pInt-Gal4)^+/Tg^; Tg(UAS:EGFP)^+/Tg^*] at 5 dpf. Bright-field (A) and EGFP (B) images are shown. Scale bar: 500 µm. (C) qPCR analysis for the *EGFP-P2A-kras^G12D^* transgene in the sibling controls and tumor-bearing larvae. The scores are normalized to expression of *rpl13a*. The data harbors three biological replicates. Error bars represent means±s.e.m. (D-I) Representative images of DAPI staining for transversal sections of the posterior intestine of tumor-bearing larvae and the sibling controls at 5 dpf. DAPI (D,E) and EGFP (F,G) images are shown. In the merged images (H,I), DAPI and EGFP signals are shown in blue and green, respectively. Scale bar: 100 µm. (J) The number of EGFP- and DAPI-positive intestinal cells. The number of nuclei was manually counted from a single section per individual larva. The data harbors 7 and 11 biological replicates from tumor-bearing larvae and the sibling controls, respectively. Error bars represent means±s.e.m. Statistical significance was tested using Student's *t*-test (unpaired, one-tailed). (K-R) Representative images of fluorescent immunohistochemistry for phosphorylated histone H3 (pH3) in transversal sections of the posterior intestine of tumor-bearing larvae and the sibling controls at 5 dpf. pH3 (K,L), DAPI (M,N) and EGFP (O,P) images are shown. White arrow indicates intestinal cells positive for pH3, EGFP and DAPI. In the merged images (Q,R), pH3, DAPI and EGFP signals are shown in red, blue and green, respectively. Scale bar: 100 µm. (S) The number of intestinal cells positive for pH3, EGFP and DAPI. The number of pH3-, EGFP- and DAPI-positive cells was counted from a single section per individual larva. The data harbors 8 and 6 biological replicates from tumor-bearing larvae and the sibling controls, respectively. Error bars represent means±s.e.m. Data are representative of at least two independent experiments.

*pInt-Gal4* expression, judged by EGFP expression, was detectable from late 4 dpf (days post-fertilization) (Fig. 2A,B). Outgrowth of posterior intestinal cells by *pInt-Gal4*-driven *kras^G12D^* expression was evident at 5 dpf (Fig. 2A,B). Oncogene expression was confirmed by quantitative real-time PCR (qPCR) (Fig. 2C and Table S1). Moreover, 100% of larvae harboring both *pInt-Gal4* and *5×UAS:EGFP-P2A-kras^G12D^* exhibited the outgrowth phenotype at 5 dpf (Fig. S2A). Thus, at this stage, we were able to phenotypically discriminate tumor-bearing larvae. The number of intestinal cells determined by DAPI staining in *kras^G12D^*-expressing larvae was significantly increased compared to that in the controls expressing EGFP under regulation by *pInt-Gal4* (Fig. 2D-J). In the previous study, Wallace et al. show that the mitotic rate of intestinal epithelial cells is high (∼40%) through 3 dpf, dropping at ∼4-5 dpf (<5%) (Wallace et al., 2005). Despite the assumption that the majority of intestinal cells are post-mitotic at 5 dpf, we counted the number of mitotic cells by pH3 (phosphorylated histone H3) staining (Fig. 2K-S) and BrdU-incorporation experiments at this time point (Fig. S2B-J). The number of pH3-positive mitotic cells (Fig. 2K-S) and BrdU-incorporated cells (Fig. S2B-J) was consistently higher in *kras^G12D^*-expressing larvae than in the sibling controls, strongly suggesting that *pInt-Gal4*-driven *kras^G12D^* expression promoted mitosis of intestinal cells.

**Fig. 3.**
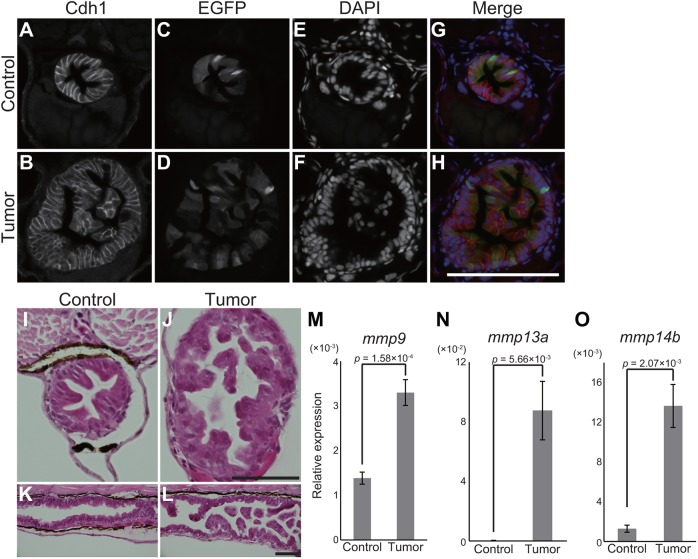
***pInt-Gal4* driven *kras^G12D^* expression results in intestinal epithelial tumor.** (A-H) Representative images of fluorescent immunohistochemistry for Cdh1 in transversal sections of the posterior intestine of the sibling controls and tumor-bearing larvae at 5 dpf. Cdh1 (A,B), EGFP (C,D) and DAPI (E,F) images are shown. In the merged images (G,H), Cdh1, EGFP and DAPI signals are shown in red, green and blue, respectively. Scale bar: 100 µm. (I-L) Representative images of HE-stained intestinal sections of the sibling controls (I,K) and tumor-bearing larvae (J,L) at 5 dpf. Transversal and sagittal sections are shown in I,J and K,L, respectively. Scale bars: 50 µm. (M-O) qPCR analysis for mmp genes in the intestine at 9 dpf. The scores are normalized to expression of *rpl13a*. The data harbors 5 biological replicates, each containing the intestines from 5 larvae. Error bars represent means±s.e.m. Statistical significance was tested using Student's *t*-test (unpaired, one-tailed). Data are representative of at least two independent experiments.

Upon closer examination of *kras^G12D^*-expressing posterior intestine, we found that *pInt-Gal4* was expressed in Cdh1 (E-cadherin)-positive intestinal cells (Fig. 3A-H), indicating that expression of *pInt-Gal4* occurred specifically in epithelial cells in the posterior intestine. Fig. 3A-H demonstrates that intestinal epithelial cells outgrew apically, whereas the basal membrane structure seemed unaffected, with hematoxylin and eosin (HE) staining supporting these findings (Fig. 3I-L). Despite the disorganized structure of the posterior intestine, the intestinal lumen was not completely disrupted (Fig. 3I-L). Consistent with this, food was present in the intestinal lumen of tumor-bearing larvae following feeding (Fig. S3A,B).

We did not observe visible invasion and dissemination of EGFP-positive cells in our experimental window (Fig. 3A-H and data not shown). Despite this, qPCR experiments and *in situ* hybridization demonstrated that expression of matrix metalloproteinase genes (*mmp9*, *mmp13a* and *mmp14b*) was strongly increased in *kras^G12D^*-expressing intestinal cells, a molecular clue for invasiveness of tumor cells (Fig. 3M-O and Fig. S4) (Hanahan and Weinberg, 2011). Altogether, these suggest that the detected outgrowth of intestinal epithelial cells resulted in formation of intestinal tumor. According to the histological definitions for malignant tumor (cancer), lack of invasion and metastasis implicate that the intestinal tumor might be benign. However, because our following analyses revealed systemic adverse effects on the host by the intestinal tumor, in this article we simply define our model as an intestinal tumor model. Collectively, we found a combination of the *Gal4* line and oncogene that drives the intestinal tumor at an early stage of zebrafish development.

### Zebrafish intestinal tumor causes local and distant inflammation

In addition to the classical definitions for cancer (malignant tumor), recent advances in molecular biology have revealed a set of molecular features that is useful to characterize cancer, known as the hallmarks of cancer (Hanahan and Weinberg, 2011). For example, it is known that cancer recruits innate immune cells such as neutrophils for survival and for promoting metastasis, and that cancer causes systemic, distant inflammation, phenomena observed across species, including in human patients (Fearon et al., 2012; Hanahan and Weinberg, 2011; McAllister and Weinberg, 2014). Importantly, zebrafish models have played important roles in this field, providing significant insights into the dynamics of innate immune cells in processes such as tumor initiation *in vivo* (Feng et al., 2012, 2010; Mione and Zon, 2012; Patton, 2012). In order to determine whether the intestinal tumor recruits neutrophils and causes systemic inflammation, we generated tumor-bearing larvae carrying *Tg(lyz:EGFP)*, which expresses EGFP in neutrophils (Kitaguchi et al., 2009).

**Fig. 4.**
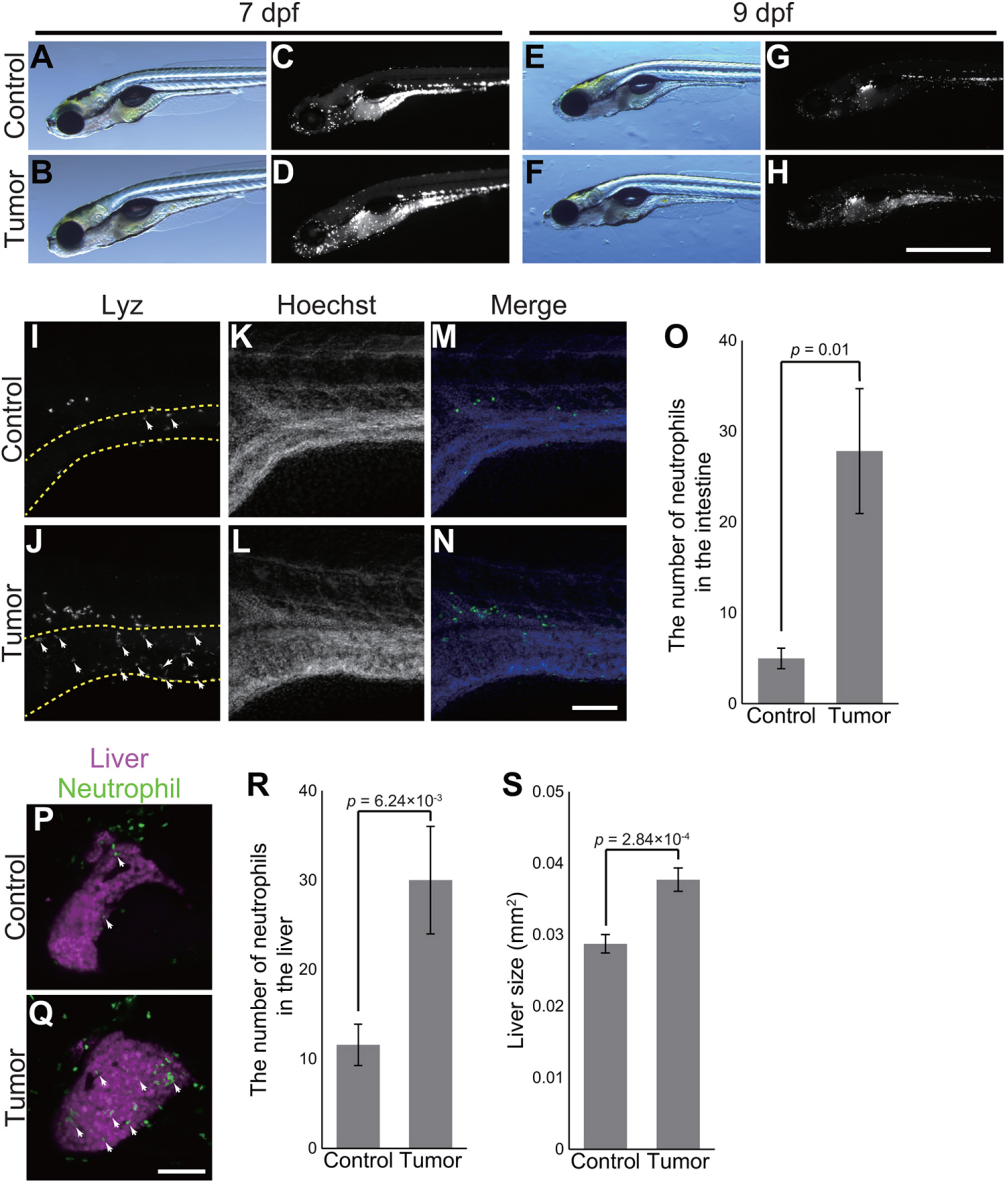
**The zebrafish intestinal tumor instigates local and distant inflammation.** (A-H) Representative images of the sibling controls and tumor-bearing larvae carrying *Tg(lyz:EGFP)* transgene at 7 and 9 dpf. Bright-field (A,B,E,F) and EGFP (C,D,G,H) images are shown. Scale bar: 1 mm. (I-N) Representative images of whole-mount fluorescent immunohistochemistry for Lyz in the intestines of the sibling controls and tumor-bearing larvae at 7 dpf. Lyz (I,J) and Hoechst 33342 (K,L) images are shown. (I,J) The intestine is shown by dashed yellow lines. (M,N) In the merged images, Lyz and Hoechst 33324 signals are shown in green and blue, respectively. White arrows indicate representative neutrophils in the intestine. Scale bar: 100 µm. (O) The number of neutrophils in the intestines of the sibling controls and tumor-bearing larvae. The data harbors 6 biological replicates. Error bars represent means±s.e.m. Statistical significance was tested using Student's *t*-test (unpaired, one-tailed). (P,Q) Representative images of the livers of the sibling controls and tumor-bearing larvae carrying *Tg(lyz:EGFP)* and *Tg(fabp10a:mCherry)* at 7 dpf. Neutrophils and the liver are shown by green and magenta, respectively. White arrows indicate representative neutrophils in the liver. Scale bar: 100 µm. (R) The number of neutrophils in the livers of the sibling controls and tumor-bearing larvae at 7 dpf. The data harbors 12 biological replicates. Error bars represent means±s.e.m. Statistical significance was tested using Student's *t*-test (unpaired, one-tailed). (S) Liver size of the sibling controls and tumor-bearing larvae at 7 dpf. Liver size was measured from *Tg(fabp10a:mCherry)* images using ImageJ software. The data harbors 12 biological replicates. Error bars represent means±s.e.m. Statistical significance was tested using Student's *t*-test (unpaired, two-tailed). Data are representative of at least two independent experiments.

Microscopic analyses showed a considerable increase in the number of neutrophils at the whole-organism level in tumor-bearing larvae at 7 dpf (Fig. 4A-H). Immunostaining with anti-Lyz antibody revealed that neutrophils were accumulated in the intestinal tumor when compared to the normal intestine (Fig. 4I-O). During the course of the experiments, we noted that neutrophils had also infiltrated the liver (Fig. 4P-R). In order to better visualize tumor-induced liver inflammation, mCherry was expressed specifically in the liver using the liver-specific *fabp10a* promoter [*Tg(fabp10a:mCherry)*] (Fig. 4P-R) (Her et al., 2003). We counted the number of EGFP-positive neutrophils in the liver expressing mCherry. As a result, we found that the number of neutrophils in the liver of tumor-bearing larvae was greater than that in the sibling controls (30±6.0 vs 12±2.3, respectively, *P*=0.0062; Fig. 4P-R). With respect to local and systemic inflammation, the intestinal tumor we developed appeared to harbor a feature of cancer (malignant tumor). It is also possible that abnormal proliferation of intestinal cells driven by *kras^G12D^* disrupts intestinal barrier function, contributing to systemic inflammation.

Furthermore, the livers of tumor-bearing larvae were larger than those of their sibling controls, a phenomenon known as hepatomegaly (0.038±0.00016 vs 0.028±0.0013 mm^2^, respectively, *P*=0.00016; Fig. 4S). Tumor-induced hepatomegaly is also seen in mammalian tumor models, including a colon cancer model (Bonetto et al., 2016; Hojo et al., 2017), and human cancer patients (Lieffers et al., 2009). These results suggest that the intestinal tumor adversely affects the liver, and that the model is able to recapitulate tumor-induced phenotypes observed in mammals and human patients.

Although we did not observe the EGFP signal in the liver of tumor-bearing larvae (Fig. S5A-D), there was a possibility that *pInt-Gal4* was expressed in the liver at a very low level and resultant expression of *kras^G12D^* caused hepatomegaly. To address this, we first analyzed expression of *EGFP-P2A-kras^G12D^* mRNA in the livers of sibling controls and tumor-bearing larvae. Our data demonstrated that expression of *EGFP-P2A-kras^G12D^* mRNA was very low (cycle threshold was typically >35) and that the scores were similar between the sibling controls and tumor-bearing larvae (Fig. S5E). Moreover, even overexpression of *kras^G12D^* in the liver using *Tg(Liver-Gal4)* did not enlarge the liver at 7 dpf (Fig. S5F-H). Based on these results, we concluded that the observed liver phenotypes were caused by the distant intestinal tumor.

### Zebrafish intestinal tumor impedes organismal growth and causes organismal death

**Fig. 5.**
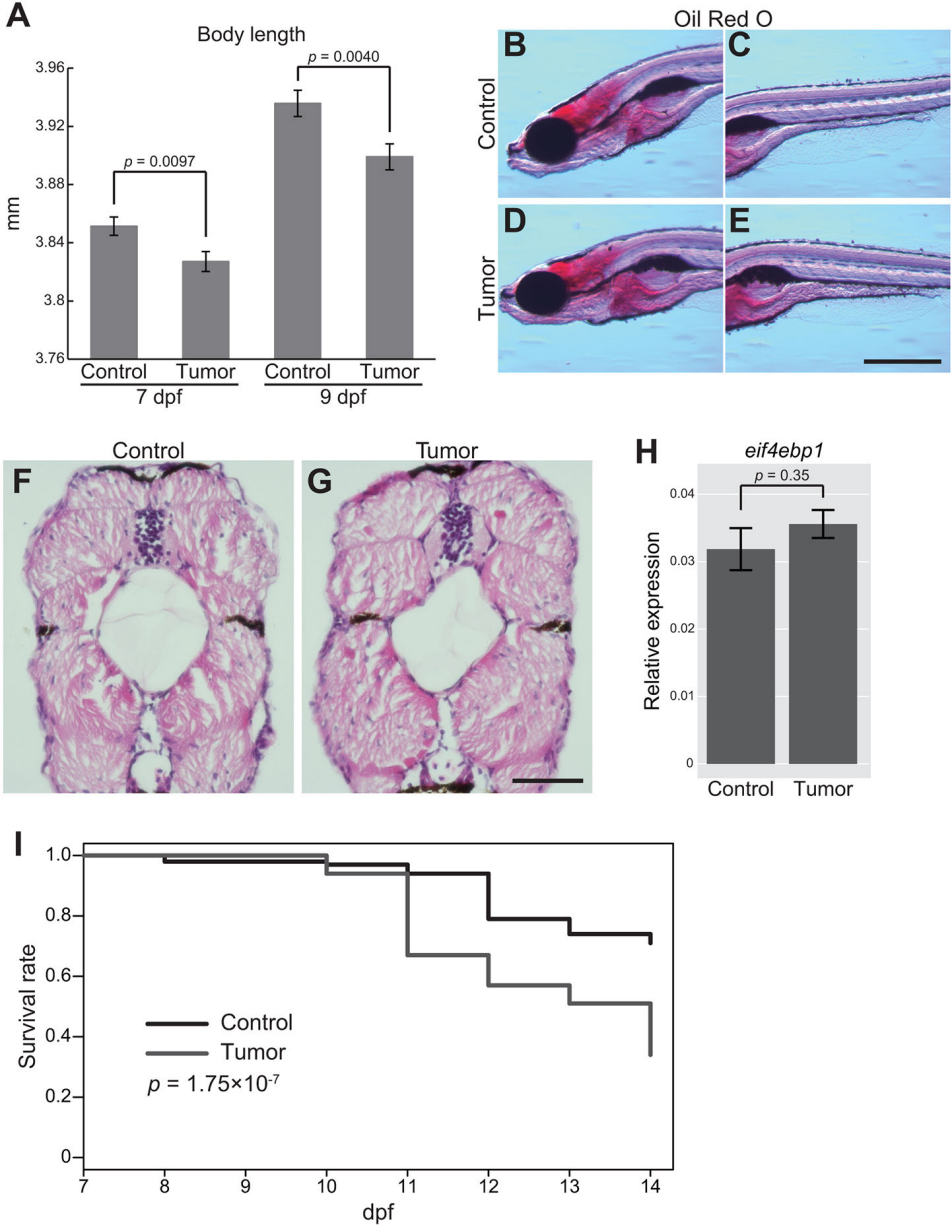
**The zebrafish intestinal tumor causes the systemic growth defect and organismal death.** (A) Body length data of the sibling controls and tumor-bearing larvae at 7 and 9 dpf. The number of larvae used is 163 (7 dpf control larvae), 155 (7 dpf tumor-bearing larvae), 154 (9 dpf control larvae) and 154 (9 dpf tumor-bearing larvae). Data from three independent clutches are pooled. Data from each clutch are shown in Fig. S6. Error bars represent means±s.e.m. Statistical significance was tested using Student's *t*-test (unpaired, two-tailed). (B-E) Representative images of Oil Red O staining for the sibling controls (B,C) and tumor-bearing larvae (D,E) at 9 dpf. Scale bar: 500 µm. Red-stained areas represent total lipids in larvae. (F,G) Representative images of HE-stained transversal body sections for the sibling controls (F) and tumor-bearing larvae (G) at 9 dpf. Scale bar: 50 µm. (H) qPCR analysis for *eif4ebp1* in the body (without the intestine or intestinal tumor) in the sibling controls and tumor-bearing larvae at 9 dpf. Scores are normalized to expression of *rpl13a*. The data harbors 5 biological replicates, each containing 5 larvae. Error bars represent means±s.e.m. Statistical significance was tested using Student's *t*-test (unpaired, two-tailed). (I) Survival rates of the sibling controls and tumor-bearing larvae from 7 to 14 dpf are shown by the Kaplan–Meier curve. Data were pooled from three independent experiments. The total number of analyzed larvae is 100 each. Statistical significance was tested using the log-rank test. Data are representative of at least two independent experiments, except I.

Next, to further demonstrate utility of the novel intestinal tumor model, we aimed to identify other systemic effects caused by the intestinal tumor. We found that tumor-bearing zebrafish larvae were significantly smaller than the sibling controls (Fig. 5A and Fig. S6), the difference observable from 7 dpf. The results varied among clutches at 7 dpf, whereas the growth defect phenotype was very consistent at 9 dpf (Fig. S6). The growth defect phenotype was identified in the complete absence of food (i.e. exogenous nutrient): although zebrafish larvae are able to eat from around 5 to 6 dpf, yolk-derived nutrients inherited from the mother keep fish alive without visible abnormalities at least until 9 dpf. This enabled us to ignore experimental variations on zebrafish behaviors related to eating and on nutrient absorption rate in the intestine in explaining the growth defect phenotype. Based on these analyses, we concluded that the local intestinal tumor caused a systemic growth defect.

It is well known that tumor-bearing animals waste muscle and fat, resulting in a loss of weight (i.e. tumor-induced cachexia) (Das et al., 2011; Fearon et al., 2012; Figueroa-Clarevega and Bilder, 2015; Kwon et al., 2015). In fact, Kwon et al. found that fly tumors alter homeostasis of systemic lipids, including triglyceride (TG) (Kwon et al., 2015). To explore whether the growth defect phenotype could be attributed to cachexia, Oil Red O staining for neutral TGs and lipids was performed. Strong staining was detected for the liver and brain at 9 dpf, a pattern that was not prominently different between tumor-bearing larvae and the sibling controls (Fig. 5B-E). This suggested that the intestinal tumor at this stage did not have a strong impact on the systemic lipid level. In addition, HE staining did not find obvious loss of host tissues such as muscle at 9 dpf (Fig. 5F,G). These were consistent with qPCR data showing that *eif4ebp1* expression, a marker for reduced insulin signaling (Figueroa-Clarevega and Bilder, 2015; Kwon et al., 2015), was not affected by the intestinal tumor (Fig. 5H). Thus, the growth defect phenotype we identified was unlikely to be canonical cachexia (Figueroa-Clarevega and Bilder, 2015; Kwon et al., 2015).

Next, we asked whether the intestinal tumor increases mortality of zebrafish. We counted the number of dead and live larvae every day and found that the survival rate of tumor-bearing larvae (less than 50% at 14 dpf) was significantly lower than that of the sibling controls (approximately 80%; Fig. 5I). This phenotype was not due to a complete defect in swimming ability and/or a complete loss of appetite in tumor-bearing larvae, because tumor-bearing larvae were able to swim and eat (Fig. S3A,B). Importantly, visible metastases were still not detected by microscopic inspection at 14 dpf (data not shown), indicating that the local intestinal tumor affected the survival rate.

Taken together, the intestinal tumor driven by strong oncogene *kras^G12D^* expression was histologically classified as benign, yet it was detrimental for organismal physiology, causing inflammation, hepatomegaly, growth defects and organismal death. Practically, our novel intestinal tumor model is useful in that the major systemic phenotypes, which are clinically observed, occur within 2 weeks after fertilization, when zebrafish larvae are still small and transparent.

### Zebrafish intestinal tumor lowers bile alcohol synthesis

**Fig. 6.**
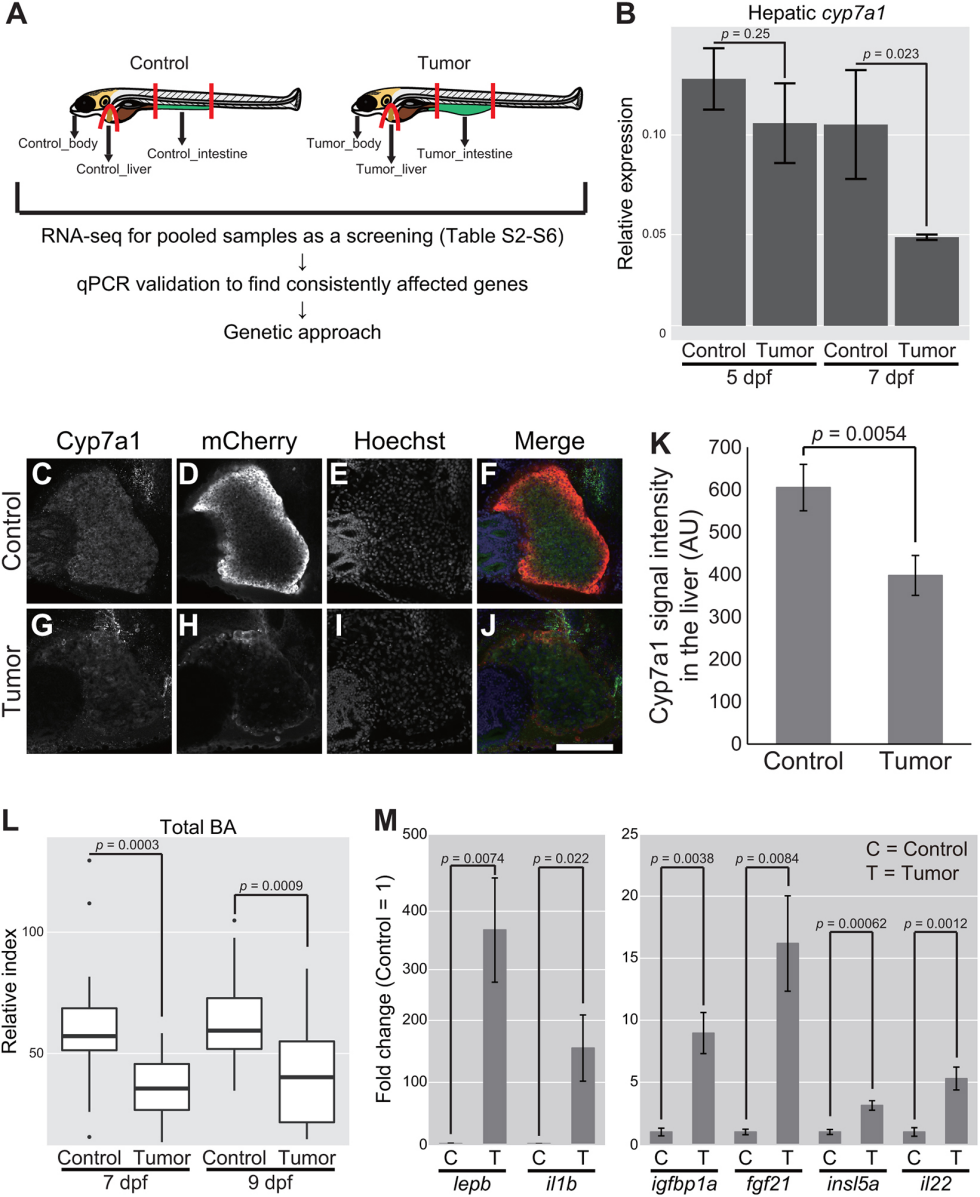
**Whole-organism gene expression analysis identifies tumor–liver crosstalk characterized by hepatic *cyp7a1* and total BA.** (A) Schematic representation of zebrafish dissection in our RNA-seq experiments followed by qPCR validation and genetic approaches. (B) Expression of *cyp7a1* in the liver. The scores are normalized to expression of *rpl13a*. The data harbors 3 biological replicates, each containing 7 larvae for 5 dpf and 5 larvae for 7 dpf, respectively. Error bars represent means±s.e.m. Statistical significance was tested using Student's *t*-test (unpaired, one-tailed). (C-J) Representative images of whole-mount fluorescent immunohistochemistry for Cyp7a1 in the livers of the sibling controls and tumor-bearing larvae carrying *Tg(fabp10a:mCherry)* at 7 dpf. Cyp7a1 (C,G), mCherry (D,H) and Hoechst 33342 (E,I) images are shown. In the merged images (F,J), Cyp7a1, mCherry and Hoechst signals are shown in green, red and blue, respectively. Scale bar: 100 µm. (K) The averages for Cyp7a1 signal intensity in the liver of the sibling controls and tumor-bearing larvae at 7 dpf are shown. The number of larvae used is 14 and 11 for the sibling controls and tumor-bearing larvae, respectively (one independent experiment). Error bars represent means±s.e.m. Statistical significance was tested using Student's *t*-test (unpaired, one-tailed). (L) Measurement for systemic BA levels at 7 and 9 dpf. The number of larvae used is 19 (7 dpf control larvae), 19 (7 dpf tumor-bearing larvae), 22 (9 dpf control larvae) and 18 (9 dpf tumor-bearing larvae). The scores are relative index determined using bile acids as standards (see Materials and Methods). Statistical significance was tested using Student's *t*-test (unpaired, one-tailed). (M) Expression of a set of secreted protein-coding genes in the intestinal tumor and normal intestine. The scores are normalized to expression of *rpl13a* and to the sibling controls (=1). The data harbors 5 biological replicates, each containing 5 larvae. Error bars represent means±s.e.m. Data are representative of at least two independent experiments, except C-K.

To examine the effects of the intestinal tumor on the host at the gene expression level and identify differentially expressed genes (DEGs), whole-organism RNA sequencing (RNA-seq) experiments were performed. Zebrafish at 7 dpf were roughly dissected into three parts: the liver, the intestinal tumor or normal intestine, and the rest of the body (Fig. 6A and Tables S2-S6). We were particularly focused on the liver since the liver was preferentially inflamed by the intestinal tumor (Fig. 4), despite a lack of visible metastasis to the liver in our experimental setting. A set of genes potentially affected by the intestinal tumor (Tables S2-S6) was used for further validation by qPCR to identify consistently affected genes: RNA-seq experiments served as a screening to find candidate DEGs.

Notably, we found that hepatic *cyp7a1*, the gene encoding the rate-limiting enzyme that acts at the first step of converting cholesterol to bile acids, in the case of zebrafish, BA (Kuipers et al., 2014; Reschly et al., 2008; Thomas et al., 2008), tended to be reduced in the presence of the intestinal tumor (Fig. 6B and Tables S2-S6). This observation was further validated at the protein level (Fig. 6C-K). Although the extent of reduction for the amount of Cyp7a1 protein was relatively mild, total BA was individually quantified, and larvae from multiple clutches were analyzed to test whether the reduced amount of Cyp7a1 protein resulted in a consequent drop in BA. The colorimetric quantitative assay demonstrated that total BA levels were significantly reduced (∼50%) in tumor-bearing larvae compared with sibling controls both at 7 dpf and 9 dpf (Fig. 6L). As expected, deletion of *cyp7a1* abolished BA production (Fig. S7A-G). We also noted that reduction in total BA levels was more robust than alteration in expression of *cyp7a1*, and that total BA levels were already reduced at 5 dpf (Fig. S7H). Despite the reduction in total BA levels, total cholesterol levels were not significantly affected by the intestinal tumor (Fig. S7I). These data suggested that the zebrafish intestinal tumor disrupts hepatic BA synthesis possibly via *cyp7a1* in the liver, an anomaly that could account for the systemic phenotypes caused by the intestinal tumor.

We then examined expression of genes known to be involved in BA homeostasis and/or targets of the BA pathway. Data revealed that genes encoding bile transporters (*slc10a2* and *slc10a4*), the G protein-coupled bile acid receptor (*gpbar1*), Farnesoid X receptors (FXRs; *nr1h4* and *nr1h5*) and known FXR targets (*insig2* and *apoa1b*) (Wang et al., 2008) responded differently to the intestinal tumor. This suggested that bile homeostasis in tumor-bearing larvae was rewired in a complex manner (Fig. S8A,G).

We next analyzed our RNA-seq data on the normal intestine and the intestinal tumor. Comparison between these two samples identified a set of genes strongly elevated in the intestinal tumor (Fig. 6M and Fig. S9A,B). DEGs included inflammatory response genes, including *interleukin 1b* (*il1b*) and *matrix metallopeptidase 13a* (*mmp13a*), and *myeloid-specific peroxidase* (*mpx*; a marker for neutrophils and macrophages), which were in line with our imaging data (Fig. 4I-O), and known RAS targets such as *gamma-glutamyltranspeptidase1* (*ggt1*) (Fig. S9A,B). Moreover, several secreted factors were elevated, including *leptin b* (*lepb*), *insulin-like growth factor binding protein 1a* (*igfbp1a*), *insulin-like peptide 5 a/b* (*insl5a* and *b*), *fibroblast growth factor 21* (*fgf21*), *interleukin 22* (*il22*) and *il1b* (Fig. 6M). Genes encoding secreted proteins that were upregulated in the intestinal tumor were considered as promising candidates that may reduce the production of hepatic BA and/or underlie the systemic phenotypes. Fgf19 and Fgf21 in mice have a role in controlling bile acid synthesis (Degirolamo et al., 2016). The insulin antagonist ImpL2 causes cachexia in *Drosophila*, and IGFBPs have been implicated in mammalian cancers (Baxter, 2014; Figueroa-Clarevega and Bilder, 2015; Kwon et al., 2015). *insl5* encodes a peptide that belongs to a relaxin family, as does fly Dilp8 (Burnicka-Turek et al., 2012; Grosse et al., 2014). Mouse studies reported a role for Insl5 in glucose homeostasis and orexigenic signaling, but its function in tumor-associated pathology is unknown (Burnicka-Turek et al., 2012; Grosse et al., 2014). It is also possible that inflammatory cytokines such as *il1b* and *tnf* reduce expression of hepatic *cyp7a1* (Okin and Medzhitov, 2016). Overall, the whole-animal-level RNA-seq experiments and qPCR revealed the intriguing abnormality in liver metabolism coincident with deregulated expression of secreted-protein-coding genes in the intestinal tumor.

### Driving *cyp7a1* expression in the liver ameliorates tumor-induced liver inflammation

**Fig. 7.**
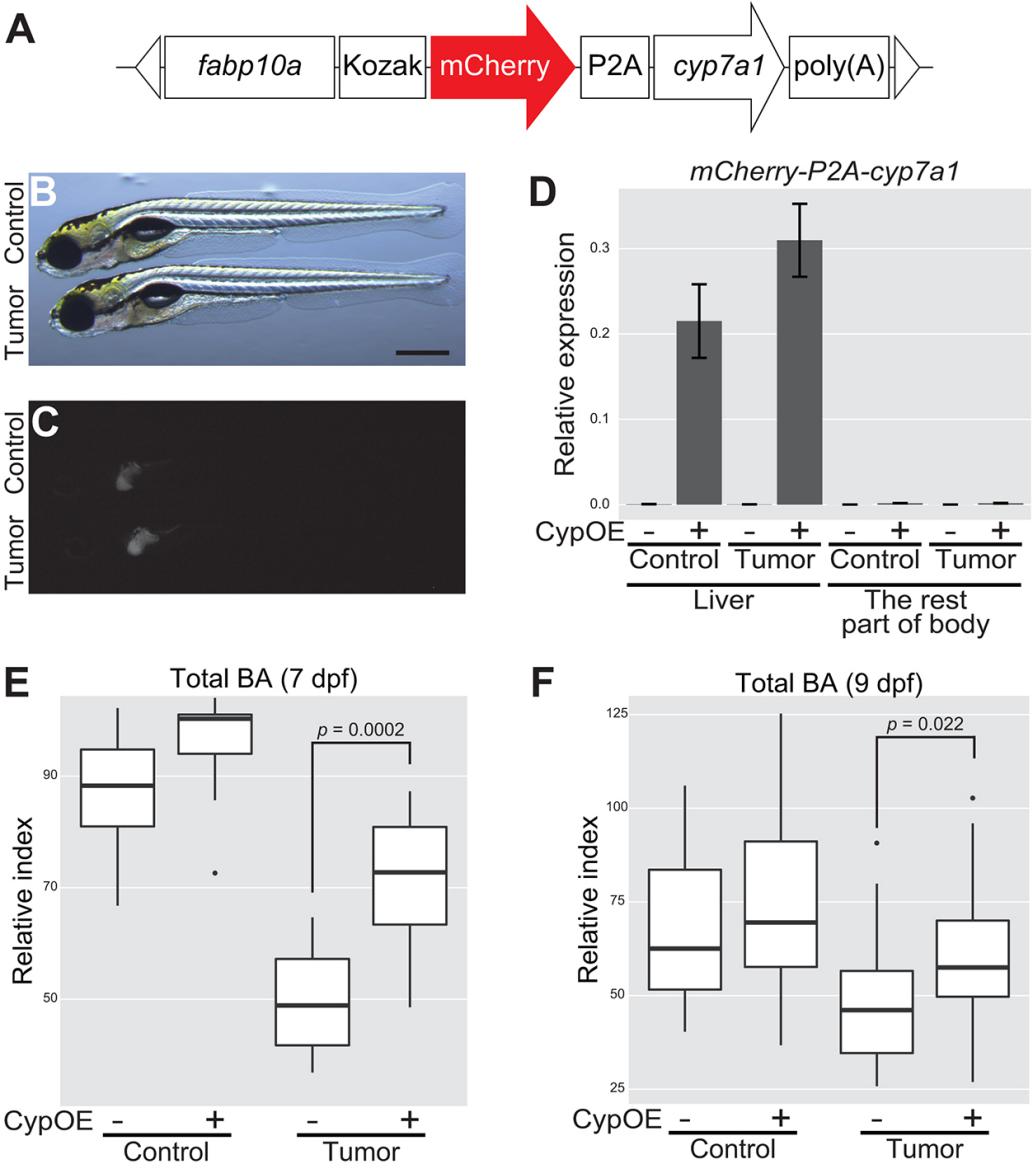
**Overexpression of *cyp7a1* in the liver restores the amount of total BA in tumor-bearing larvae.** (A) The structure of *fabp10a:mCherry-P2A-cyp7a1*. The white triangles represent the recognition sequence by I-*Sce*I meganucleases. (B,C) Representative images of *mCherry-P2A-cyp7a1* transgene expression in the liver. Control refers to *Tg(pInt-Gal4)^+/Tg^;* *Tg(UAS:EGFP)^+/Tg^;* *Tg(fabp10a:mCherry-P2A-cyp7a1)^+/Tg^*, and tumor-bearing larvae to *Tg(pInt-Gal4)^+/Tg^;* *Tg(5×UAS:EGFP-P2A-kras^G12D^)^+/Tg^;* *Tg(fabp10a:mCherry-P2A-cyp7a1)^+/Tg^*. Scale bar: 500 µm. Bright-field (B) and mCherry (C) images are shown. (D) qPCR analysis for detecting *mCherry-P2A-cyp7a1* mRNA in the liver and the rest of the body at 7 dpf. The scores are normalized to expression of *rpl13a*. The data harbors 3 biological replicates, each containing 3 larvae. Error bars represent means±s.e.m. CypOE – and + indicate the absence and presence of *Tg(fabp10a:mCherry-P2A-cyp7a1)*, respectively. (E,F) Measurement for systemic BA levels at 7 (*n*=10 per a group) and 9 (*n*=30-31 per a group) dpf. The scores are relative index determined using bile acids as standards (see Materials and Methods). Statistical significance was tested using Student's *t*-test (unpaired, one-tailed). Data are representative of at least two independent experiments.

In order to investigate whether the altered *cyp7a1* expression in the liver affects tumor-induced systemic phenotypes, we generated a transgenic line expressing *cyp7a1* under the control of the *fabp10a* promoter (Her et al., 2003). Expression of *cyp7a1* was linked to mCherry with P2A (Kim et al., 2011) (Fig. 7A). Transgene expression was ascertained by microscopic observation and qPCR (Fig. 7B-D). Overexpression of *cyp7a1* in the liver significantly restored total BA levels both at 7 and 9 dpf in tumor-bearing larvae (Fig. 7E,F). The transgene also tended to increase total BA in their tumor-free sibling controls. Altogether, the *fabp10a:mCherry-P2A-cyp7a1* transgene was able to restore BA production in tumor-bearing larvae, further supporting that the intestinal tumor affects cholesterol–BA flux via *cyp7a1*.

**Fig. 8.**
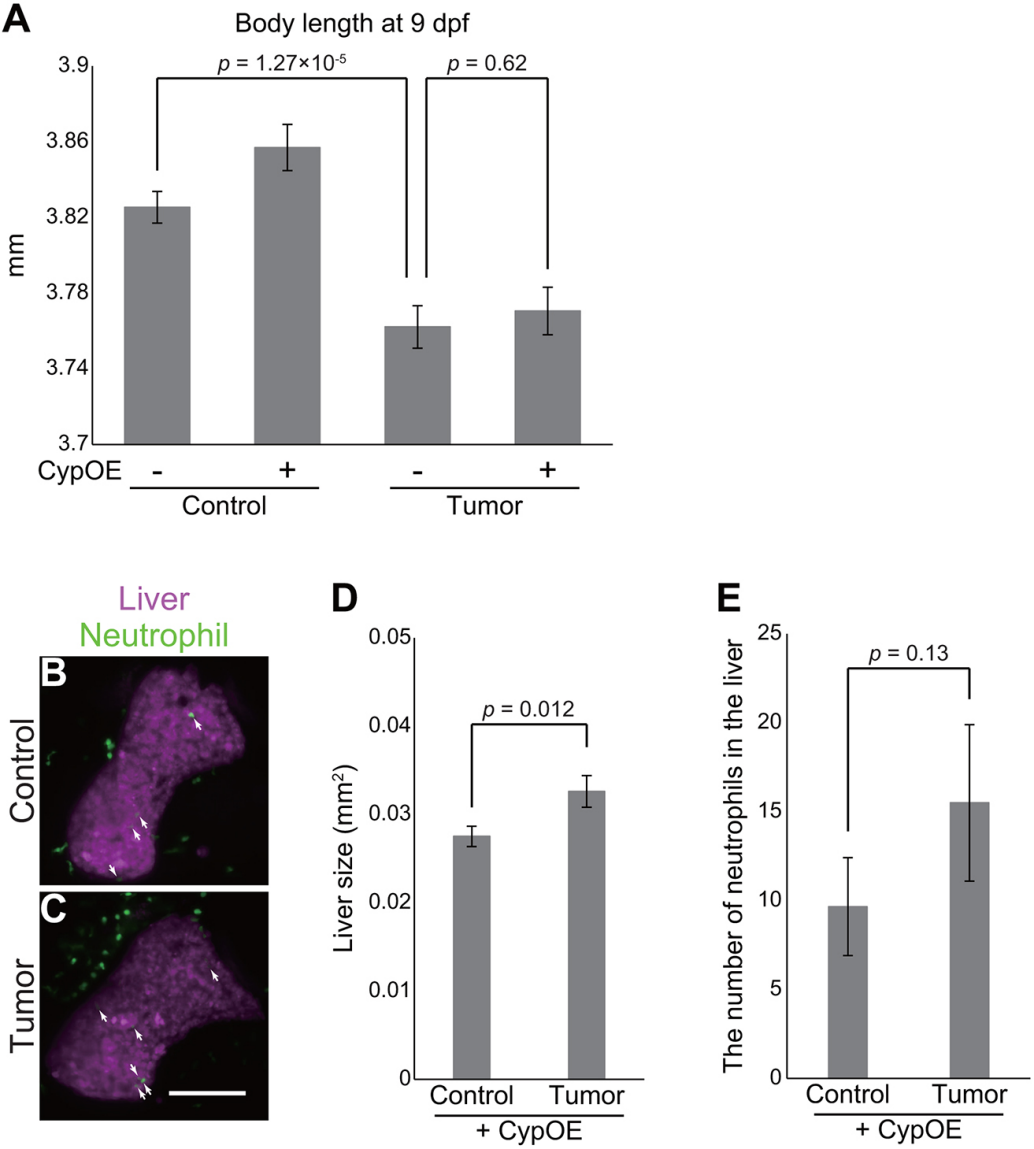
**Overexpression of *cyp7a1* in the liver ameliorates tumor-induced liver inflammation.** (A) Body length data of the sibling controls and tumor-bearing larvae at 9 dpf in the *Tg(fabp10a:mCherry-P2A-cyp7a1)* background. The number of larvae used is 79 (control larvae), 73 [control larvae with *Tg(fabp10a:mCherry-P2A-cyp7a1)*], 81 (tumor-bearing larvae) and 74 [tumor-bearing larvae with *Tg(fabp10a:mCherry-P2A-cyp7a1)*]. Error bars represent means±s.e.m. Statistical significance was tested using Student's *t*-test (unpaired, two-tailed). CypOE − and + indicate the absence and presence of *Tg(fabp10a:mCherry-P2A-cyp7a1)*, respectively. (B,C) Representative images of the livers of the sibling controls and tumor-bearing larvae carrying *Tg(lyz:EGFP)* and *Tg(fabp10a:mCherry-P2A-cyp7a1)* at 7 dpf. Neutrophils and the liver are shown by green and magenta, respectively. Scale bar: 100 µm. White arrows indicate representative neutrophils. (D) Liver size and (E) the number of neutrophils of the sibling controls and tumor-bearing larvae carrying *Tg(lyz:EGFP)* and *Tg(fabp10a:mCherry-P2A-cyp7a1)* at 7 dpf. Liver size was measured from *Tg(fabp10a:mCherry-P2A-cyp7a1)* images using ImageJ software. The data harbors 18 biological replicates (pooled from two independent experiments). Error bars represent means±s.e.m. Statistical significance was tested using Student's *t*-test (unpaired, one-tailed). Data are representative of at least two independent experiments, except D,E.

These results promoted us to test whether overexpression of *cyp7a1* in the liver could rescue the intestinal-tumor-induced systemic phenotypes. We examined whether three major tumor-induced phenotypes, liver inflammation, hepatomegaly and the growth defect, were rescued by the *fabp10a:mCherry-P2A-cyp7a1* transgene (Fig. 8). We found that *cyp7a1* overexpression did not significantly rescue the growth defect phenotype (Fig. 8A). As was the case in Fig. 5A, the results to some extent varied depending on clutches: in one clutch, we observed a trend for the rescue, but not in a different clutch. Upon pooling data from multiple clutches, we concluded that *cyp7a1* overexpression did not consistently and significantly rescue the growth defect phenotype. Moreover, tumor-induced hepatomegaly [0.028±0.0013 mm^2^ (control) vs 0.038± 0.0016 mm^2^ (tumor), *P*=0.00016: Fig. 4S] was not affected by *cyp7a1* overexpression in the liver [0.028±0.0011 mm^2^ (control) vs 0.033±0.0018 mm^2^ (tumor), *P*=0.012: Fig. 8B-D].

Interestingly, the number of neutrophils observed in the liver was comparable between the sibling controls and tumor-bearing larvae in the *Tg(fabp10a:mCherry-P2A-cyp7a1)* background [9.7±2.8 (control) vs 16±4.4 (tumor), *P*=0.134: Fig. 8B,C,E], in contrast to our data in the *Tg(fabp10a:mCherry)* background [12±2.3 (control) vs 30±6.0 (tumor), *P*=0.0062: Fig. 4P-R]. As an important detail, these experiments (Figs 4R,S and 8B-E) were performed using staged-matched larvae (7 dpf), which was demonstrated by the fact that liver size and the number of neutrophils were similar in the control groups. Despite statistical insignificance, there was still a trend for the increase in the number of neutrophils in tumor-bearing larvae in the *Tg(fabp10a:mCherry-P2A-cyp7a1)* background. This might suggest that the rescue by *Tg(fabp10a:mCherry-P2A-cyp7a1)* was partial, consistent with the fact that the extent of rescue for total BA levels was not 100% (Fig. 7E,F). Alternatively, another factor might contribute to liver inflammation by the intestinal tumor. We also measured whole-body expression of the *lyz* gene in the above-described genetic backgrounds, which likely reflects the total number of neutrophil (Fig. S9C). Data demonstrated that *Tg(fabp10a:mCherry-P2A-cyp7a1)* did not lower expression of *lyz* in tumor-bearing larvae. Thus, amelioration for tumor-induced inflammation was observed in a local rather than a systemic manner.

**Fig. 9.**
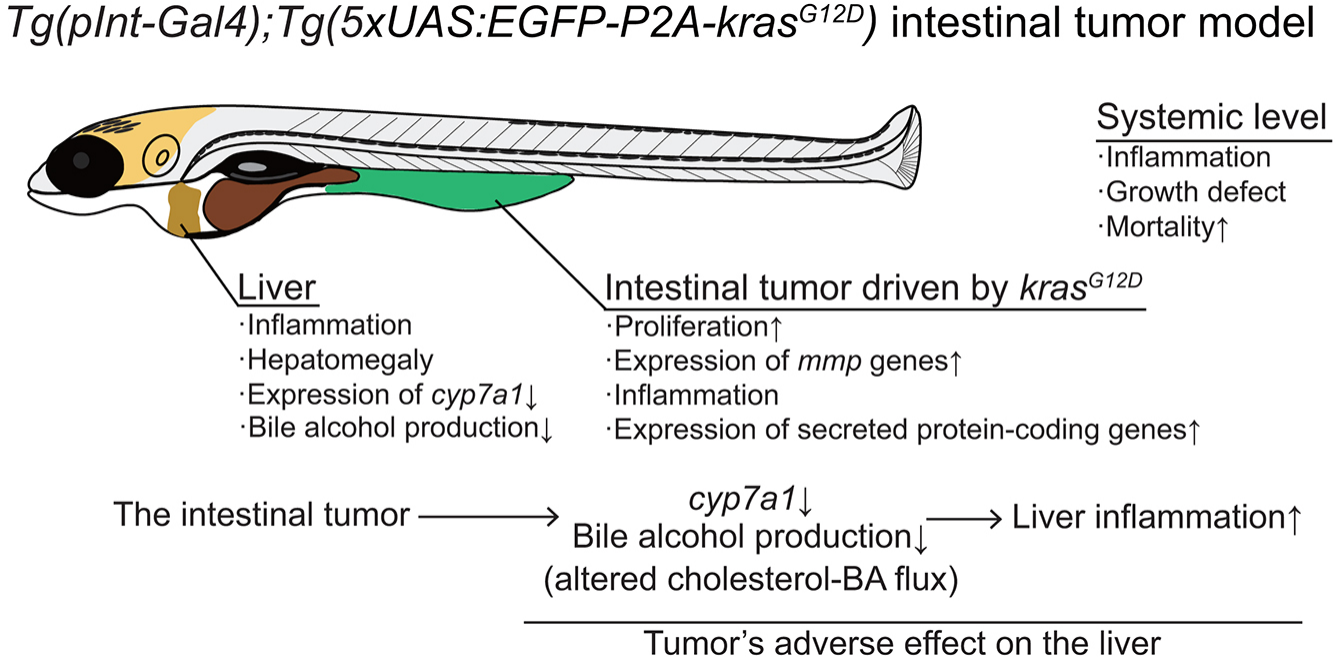
**Graphical summary of this study.** *kras^G12D^* expression driven by *pInt-Gal4* results in tumor formation in the posterior intestine. Despite being histologically benign and restricted to the intestine, the intestinal tumor causes a set of systemic adverse effects on the host. The intestinal tumor recruits neutrophils to the liver and causes hepatomegaly. Tumor-bearing larvae grow less efficiently than the sibling controls do, and die at around 14 dpf. The intestinal tumor communicates with the liver, altering cholesterol–BA flux. This interaction is important for tumor-induced liver inflammation, but not for other phenotypes.

*cyp7a1* has not been considered as a crucial host gene in tumor-induced distant inflammation. Yet, studies in different contexts support our observation that the intestinal tumor actively reduces expression of hepatic *cyp7a1* to promote liver inflammation (Fig. 9). In mice, overexpression of Cyp7a1 in the liver suppresses lipopolysaccharide (LPS)-induced hepatic inflammation and fibrosis (Liu et al., 2016). It is also known that sustained inflammation reduces expression of Cyp7a1, suggestive of a role for Cyp7a1 in inflammation in mice (Okin and Medzhitov, 2016). Collectively, the current study, as the demonstration for utility of the model, identifies *cyp7a1* as a host gene that mediates liver inflammation, one of the adverse effects on the host caused by the intestinal tumor.

## DISCUSSION

This study has two major advances. First, we established the novel zebrafish intestinal tumor model, which is suitable for studying body-wide tumor–organ interaction *in vivo*. Second, using the model, we discovered a tumor–liver interaction that mediates enhanced recruitment of neutrophils to the liver in tumor-bearing larvae via a cholesterol-metabolizing gene, *cyp7a1*, as a critical host gene.

### Establishment of a novel intestinal tumor model in zebrafish

The zebrafish intestinal tumor model we have newly established harbors several strengths for studying tumor–organ interaction at the whole-organism level (Fig. 9). The combination of *pInt-Gal4* and UAS-controlled *kras^G12D^* induces epithelial tumor formation in the posterior intestine at as early as 5 dpf, when zebrafish are small and completely transparent (Figs 1-3). Yet, zebrafish larvae after 5 dpf are able to swim and eat, and therefore it is likely that essential organs such as the liver are already mature at this time point. Even though the intestinal tumor is histologically not fully malignant, the intestinal tumor causes detrimental effects on the host, including systemic inflammation, hepatomegaly, a growth defect, metabolic defects and organismal death (Figs 4-8). The model even made it possible to visualize the intestinal tumor-induced inflammation in the liver of live larvae (Fig. 4). Furthermore, the growth defect phenotype we discovered does not depend on exogenous food intake, simplifying our investigation on how the intestinal tumor causes systemic growth defect (Fig. 5).

To date, a genetically engineered, robust zebrafish intestinal tumor model has not yet been available (Lobert et al., 2016). The structure of the intestinal tract in zebrafish is different from mice and humans, especially in that zebrafish lacks a stomach. Still, the zebrafish intestine shares common features with mammalian intestines, a notion that is validated by anatomical analysis and comprehensive gene expression study (Lobert et al., 2016; Wallace et al., 2005; Wang et al., 2010). On the basis of these reports, the zebrafish intestine appears to be analogous to the small intestine, colon and rectum of mammals. Relevance to human diseases of our model is also supported by the fact that the intestinal tumor model exhibits liver phenotypes observed in murine colon tumor models, such as *Apc^Min/^^+^*, and human patients (Bonetto et al., 2016; Lieffers et al., 2009; Narsale et al., 2015): it is of note that *Apc^Min/+^* is a model of adenoma (histologically benign) and potent in causing adverse effects on the host. Therefore, histological classification of tumors (benign or malignant) does not always correlate with the degree of adverse effects on the host. Taken together, we expect that our model will be a valuable tool for studying the biology of intestinal tumors.

It is also important to note that there are other zebrafish models that develop tumors at an early stage of zebrafish development, which are thus potentially useful for studying tumor–organ crosstalk at the whole-organism level. For instance, Mione and colleagues established a novel brain tumor model using *HRAS^V12^* in which increased brain size was observed already at 3 dpf (Mayrhofer et al., 2017). Activating the β-catenin signal promotes liver enlargement associated with enhanced proliferation at 6 dpf in the model established by Stainier and colleagues (Evason et al., 2015). These models are definitely useful to obtain insights into how various types of local tumors affect developing vertebrates.

### Identification of a tumor-induced growth defect in developing zebrafish

Our model exhibits an intriguing systemic phenotype: tumor-bearing larvae do not grow well compared to their sibling controls (Fig. 5A). This phenotype was neither accompanied with a clear reduction of the systemic lipid level (Fig. 5B-E) nor with reduced insulin signaling (Fig. 5H), common phenotypes observed in cachexia patients and animal models (Fearon et al., 2012; Figueroa-Clarevega and Bilder, 2015; Kwon et al., 2015). Hence, we at this point assume that the observed growth defect is not the typical tumor-induced cachexia.

The growth defect phenotype to some extent resembled the growth delay in flies harboring an imaginal disc tumor or local wounds (Colombani et al., 2015, 2012; Garelli et al., 2012, 2015; Katsuyama et al., 2015; Owusu-Ansah and Perrimon, 2015; Vallejo et al., 2015). Secreted fly-specific peptide Dilp8 and its receptor Lgr3 are at the core of adaptation of growth and developmental timing to local disruptions. Dilp8 interacts with Lgr3 expressed in neurons that are projected to the prothoracic gland to control biosynthesis of ecdysone, one of the master regulators for fly development (Colombani et al., 2015; Garelli et al., 2015; Vallejo et al., 2015). However, whether similar growth retardation occurs in vertebrate tumor models has not been validated. Our study demonstrates the first vertebrate model in which the local intestinal tumor impedes organismal growth. Secreted-protein-coding genes such as *insl5a* that are upregulated in the intestinal tumor may act upstream of the growth defect (Fig. 6M).

Recent advances in pediatric oncology have greatly improved the survival rate of childhood cancer patients. Importantly, it is known that survivors of childhood cancers often have ‘late complications’: long-lasting (sometimes for 40 years) complications, including growth defects (Robison and Hudson, 2014; Rose et al., 2016). Cancers by themselves and/or cancer treatments (e.g. chemotherapy) may cause late complications, but the details are still unknown. Our model develops the intestinal tumor at a juvenile stage when zebrafish larvae grow massively. The study thus points out the possibility that the local tumor could be a cause for long-lasting growth defects in human cancer patients. This can be directly addressed once we have the ability to cure the intestinal tumor in our model so that we can test whether the growth defect lasts even after removal of the intestinal tumor.

### The intestinal tumor remotely alters systemic cholesterol–BA homeostasis through *cyp7a1*-mediated tumor–liver interaction to promote liver inflammation

One of the strengths of our model is that the intestinal tumor causes systemic effects when zebrafish larvae are small enough for whole-body analysis (Fig. 9). This enabled us to perform whole-organism transcriptome analysis to capture gene expression changes in the intestinal tumor and the remaining normal organs (Fig. 6 and Tables S2-S6). We found that the liver responded to the intestinal tumor in a sensitive manner in our model (Figs 4,6). In addition to tumor-induced systemic inflammation and hepatomegaly (Fig. 4) (Egeblad et al., 2010; Fearon et al., 2012; McAllister and Weinberg, 2014), hepatic expression of *cyp7a1*, the gene encoding the rate-limiting enzyme for synthesizing bile acids/alcohol (Kuipers et al., 2014; Thomas et al., 2008), tended to be decreased at as early as 5-7 dpf in tumor-bearing larvae (Fig. 6B-K). This was concordant with the reduced total BA levels (Fig. 6L and Fig. S7H), which was not due to the decreased body size, as we did not find any correlation between body length and the amount of BA in each individual (Fig. S10A). Correlation between expression of *cyp7a1* and total BA levels can be complex because the bile–FXR signaling suppresses *cyp7a1* expression, forming a feedback loop (Wang et al., 2008).

Overexpression of *cyp7a1* in the liver by means of the *fabp10a* promoter significantly restored total BA levels in tumor-bearing larvae (Fig. 7). This indicated that *cyp7a1* expression is the rate-limiting process for maintaining normal BA levels in tumor-bearing larvae. Yet, given the marginal decrease in the amount of Cyp7a1 proteins (Fig. 6C-K), at this point, we cannot rule out the possibility that a *cyp7a1*-independent mechanism also may contribute to altered BA homeostasis.

Intriguingly, *cyp7a1* overexpression in the liver was associated specifically with buffered liver inflammation (Fig. 8): the number of neutrophils in the liver was increased in the presence of the intestinal tumor (Fig. 4P-R), which was significantly ameliorated by overexpression of *cyp7a1* in the liver (Fig. 8E). These results indicate that the intestinal tumor instigates liver inflammation through modulating expression of *cyp7a1* and cholesterol–BA flux in the liver. Given that *Tg(fabp10a:mCherry-P2A-cyp7a1)* did not rescue hepatomegaly, the growth defect or survival (Fig. S10B), liver inflammation might be independent of these phenotypes (Figs 8,9 and Fig. S10B). Our results were in line with recent studies showing a role for murine Cyp7a1 in liver inflammation in non-cancer disease models (Liu et al., 2016; Okin and Medzhitov, 2016), indicative of a generalizable role for *cyp7a1*-mediated cholesterol–BA metabolism in diseases. These reports also solidify the general utility of our novel tumor model. We emphasize that our findings are of significance in that we redefined *cyp7a1* as a host gene critical for mediating the tumor–liver–neutrophil crosstalk *in vivo*. It still remains unclear whether the decrease for total BA levels and/or altered cholesterol flux directly enhances liver inflammation, and for what the intestinal tumor causes distant inflammation in the liver. Further extensive genetic studies are ongoing to reveal the physiological significance of the altered cholesterol–BA homeostasis in tumor-bearing zebrafish larvae.

### Genetics on the physiological interaction between tumor and normal organ(s)

Here, we provide evidence for the utility of our model by showing that *cyp7a1*-mediated tumor–liver interaction underlies altered neutrophil dynamics in the livers of tumor-bearing zebrafish larvae. An importance of hepatic *cyp7a1* in the tumor's adverse effects on the host has not been previously appreciated. Thus, the study shows that our approach is powerful to uncover previously unknown contributions of ordinary genes in tumor-induced systemic phenotypes. Three major questions remain to be solved: which tumor-derived factor(s) alters *cyp7a1*-mediated cholesterol–BA flux and liver inflammation? Does *cyp7a1*-mediated liver inflammation benefit the intestinal tumor? What other host genes are responsible for the systemic tumor's adverse effects on the host in this model? We are addressing these questions by combining transcriptome and genetic experiments. Further genetic dissection on such physiologically important tumor–organ interactions will help to discover a therapy(ies) that ameliorates host physiology being harmed by tumors.

## MATERIALS AND METHODS

### Zebrafish

All animal protocols were approved by the Animal Care and Use Committee of Advanced Telecommunications Research Institute International, Japan. AB and AB/TL lines were used as standard lines. Adult fish were reared at 28°C under a 14 h/10 h light/dark cycle and fed hatched brine shrimp and the Hikari Lab 130 food (KYORIN, Japan). Fish were fed twice a day except at weekends and holidays (once a day). Embryos were obtained by mating male fish with female fish in a water tank and were maintained at 28°C in egg water (3% sea salts, 6.4 nM methylene blue) in a plastic Petri dish. Tricaine methanesulfonate (MS-222) was used as an anesthetic reagent at a concentration of 0.008% in egg water.

### Transgenic lines and plasmid construction

The transgenic zebrafish lines [gSAIzGFFD1105A (*pInt-Gal4*), gSAIzGFFM103B (*aInt-Gal4*), gSAIzGFFD886A (*Liver-Gal4*) and gSAGFF138A (*Brain-Gal4*)] were generated by *Tol2*-transposon-mediated gene trap and enhancer trap methods as described previously (Asakawa and Kawakami, 2008; Kawakami et al., 2016). *Tg(lyz:EGFP)* were obtained from the National Bioresource Project Zebrafish Core Institution under the approval of the developer (Kitaguchi et al., 2009). The constructs for generating *Tg(5×UAS:EGFP-P2A-kras^G12D^)*, *Tg(fabp10a:mCherry)* and *Tg(fabp10a:mCherry-P2A-cyp7a1)* were generated by PCR, combining the synthesized oligonucleotides and fragments amplified from the wild-type genome (Her et al., 2003; Omae et al., 2013). The sequences are provided in Table S1. Generation of *Tg(5×UAS:EGFP-P2A-kras^G12D^)* was performed as described previously (Kawakami, 2004; Thermes et al., 2002). I-*Sce*I meganuclease was purchased from New England Biolabs and used for generating *Tg(fabp10a:mCherry)* and *Tg(fabp10a:mCherry-P2A-cyp7a1)* (Thermes et al., 2002). The existence of mCherry- or EGFP-encoding transgenes was inspected using a Leica M165 FC fluorescent stereoscopic microscope (Leica).

### Screening of transgenic *Gal4* lines that can drive tumorigenesis

The *Tg(5×UAS:EGFP-P2A-kras^G12D^)* line was mated to each *Gal4* line heterozygous for the *Gal4* transgene. As an example, *Tg(5×UAS:EGFP-P2A-kras^G12D^)^+/Tg^* fish were crossed with *Tg*(*pInt-Gal4)^+/Tg^; Tg(UAS:EGFP)^+/Tg^* fish to obtain *Tg(5×UAS:EGFP-P2A-kras^G12D^)^+/Tg^; Tg(pInt-Gal4)^+/Tg^* embryos. Expression of *kras^G12D^* in the siblings was examined by EGFP expression using a Leica M165 FC fluorescent stereoscopic microscope (Leica). When larvae with a potentially tumorous phenotype were identified, larvae with no EGFP expression from the same clutch (i.e. a clutch includes siblings born on the same day from the same parents) were considered as their sibling controls. In cases where no observable phenotype could be discerned, *kras^G12D^*-expressing larvae and the sibling controls were discriminated based on genotyping experiments. In both cases, larvae harboring either *Gal4* or *Tg(5×UAS:EGFP-P2A-kras^G12D^)*, or neither, served as the sibling controls. For genotyping, genomic DNA was isolated from single larva by proteinase K (Takara; 1:100 dilution) in 10 mM Tris–HCl (pH 8.0) and 50 mM KCl and used as a PCR template. Each transgene was amplified using KAPA 2G Fast HS (NIPPON Genetics). The *tp53* genomic region was used as the PCR control. The primers used are listed in Table S1.

### Generation of *cyp7a1* mutant using the CRISPR–Cas9 system

Target sequence for CRISPR–Cas9 was searched using CRISPR direct (http://crispr.dbcls.jp/) and CHOPCHOP (http://chopchop.cbu.uib.no/). Oligonucleotide-based sgRNA transcription and *zCas9-nls* mRNA transcription were performed as described (Gagnon et al., 2014; Jao et al., 2013), respectively. sgRNA and *zCas9-nls* mRNA were microinjected into wild-type embryos at the one-cell stage. The F0 generation was reared and mated to wild-type fish to obtain the F1 generation. The F1 generation was reared and fin-clipped to extract genomic DNA followed by genotyping approximately at 1 month post-fertilization. Genomic DNA was prepared with proteinase K digestion as described above. A mutation allele was detected by high resolution melting (HRM) analysis as described (Thomas et al., 2014) and DNA sequenced (FASMAC, Kanagawa, Japan). We obtained zebrafish harboring the *cyp7a1^−5^* allele, which were kept by mating to AB/TL strain.

### RNA isolation, cDNA synthesis and qPCR

For gene expression experiments, we often pooled multiple larvae in a single tube. This was to obtain a sufficient amount of high-quality RNAs, especially when dissection was performed, and to lower the risk of selecting outliers from the clutch. Given that a single female generally produces more than 50 embryos, selecting e.g. ∼3-5 larvae from a clutch may give rise to unwanted bias in sample collection. Pooling multiple larvae and treating it as one biological replicate could be useful to reduce these risks. Total RNA was isolated using TRIzol (Thermo Fisher Scientific) or RNeasy Mini Kit (QIAGEN). In Fig. S5E, because *EGFP-P2A-kras^G12D^* is intron-less, total RNA was DNase I-treated (TaKaRa) and quantification was accompanied by qPCR against total RNA without reverse transcription. cDNA was synthesized using the SuperScript III First-Strand Synthesis System (Thermo Fisher Scientific) or Transcriptor First Strand cDNA Synthesis Kit (Roche). The obtained cDNAs were 5- or 10-fold-diluted and subjected to qPCR experiments by using the LightCycler480 Instrument II system and SYBR Green Master Mix (Roche). The obtained data were analyzed using the ΔCt method. The primers used are listed in Table S1.

### Cryosectioning and fluorescent immunohistochemistry

*Tg(5×UAS:EGFP-P2A-kras^G12D^)^+/Tg^; Tg(pInt-Gal4)^+/Tg^* and *Tg(pInt-Gal4)^+/Tg^; Tg(UAS:EGFP)^+/Tg^* larvae from the same clutch were used. At 5 dpf, larvae were collected and fixed in 4% paraformaldehyde (PFA) in PBS at 4°C overnight. Larvae were then washed with PBS five times and then embedded in 1.2% agarose and 5% sucrose in PBS. Agarose blocks were trimmed by a razor and then incubated in PBS containing 30% sucrose at 4°C overnight. After replacement with 30% sucrose solution, blocks were frozen on dry ice and stored at −80°C until cryosectioning. Larvae were transversely sectioned (thickness: 16 µm) using a Leica CM 3050 S (Leica) and sections posterior to the swimming bladder were collected (one section per individual). Cryosections were adhered on a MAS-GP typeA-coated glass slide (Matsunami Glass Ind., Ltd, Japan) and air-dried at room temperature for 30 min. Sections were rehydrated with PBS at room temperature for 30 min, and then permeabilized and blocked with 5% normal goat serum in PBS supplemented with 0.5% Triton X-100 (0.5% PBT) for 1 h. Sections were then incubated with the following primary antibodies diluted in 5% normal goat serum in 0.5% PBT at 4°C overnight: rabbit anti-phosphorylated-Histone H3 (Ser10) (pH3) (EMD Millipore, 06-570; 1:100 dilution) and rabbit anti-E-cadherin (Cdh1) (Gene Tex, GTX125890; 1:100 dilution). Sections were washed with 0.5% PBT and then incubated with secondary antibody, Alexa-Fluor-568-conjugated anti-rabbit IgG (Life Technologies; 1:400 dilution), at room temperature for 1 h. Sections were washed with 0.5% PBT and then mounted with ProLong Gold Antifade Mount with DAPI (Thermo Fisher Scientific). Fluorescent images were taken with a Nikon A1R confocal laser microscope (Nikon).

### BrdU incorporation, cryosectioning and fluorescent immunohistochemistry

*Tg(5×UAS:EGFP-P2A-kras^G12D^)^+/Tg^; Tg(pInt-Gal4)^+/Tg^* and *Tg(pInt-Gal4)^+/Tg^; Tg(UAS:EGFP)^+/Tg^* larvae from the same clutch were used. BrdU incorporation experiments were performed essentially as described previously (Takada et al., 2010). At 4 dpf, 20 larvae were transferred into egg water containing 0.5 mM bromodeoxyuridine (BrdU; Nacalai Tesque) and incubated for 24 h. At 5 dpf, larvae were rinsed with egg water and then fixed with 4% PFA in PBS. Agarose embedding and cryosectioning were performed as described above. After rehydration of cryosections by PBS, sections were treated with 2N hydrochloric acid to denature DNA at room temperature for 1 h and then washed with PBS. Blocking and antibody treatment were performed as described above. Primary antibodies, mouse anti-BrdU antibody (Developmental Studies Hybridoma Bank, G3G4; 1:500 dilution) and rabbit anti-GFP antibody (MBL, 598; 1:500 dilution), and secondary antibodies, Alexa-Fluor-568-conjugated anti-mouse IgG (Life Technologies; 1:500 dilution) and Alexa-Fluor-488-conjugated anti-rabbit IgG (Life Technologies; 1:500 dilution), were used. Sections were counterstained with Hoechst 33342 (Life Technologies; 1:2000 dilution) and mounted with 80% glycerol in PBS. Fluorescent images were taken with a Nikon A1R confocal laser microscope (Nikon).

### Whole-mount fluorescent immunohistochemistry

*Tg(5×UAS:EGFP-P2A-kras^G12D^)^+/Tg^; Tg(pInt-Gal4)^+/Tg^* and *Tg(pInt-Gal4)^+/Tg^; Tg(UAS:EGFP)^+/Tg^* larvae from the same clutch were used for staining Lyz. *Tg(5×UAS:EGFP-P2A-kras^G12D^)^+/Tg^; Tg(pInt-Gal4)^+/Tg^; Tg(fabp10a:mCherry)* and *Tg(pInt-Gal4)^+/Tg^; Tg(UAS:EGFP)^+/Tg^; Tg(fabp10a:mCherry)* larvae from the same clutch were used for staining Cyp7a1 and mCherry. At 7 dpf, larvae ware fixed in 4% PFA in PBS at 4°C overnight. Larvae were washed with PBS five times and treated with 3% hydrogen peroxide in 0.5% sodium hydride at room temperature to bleach pigments. After removing pigments, larvae ware washed with PBS, and then transferred into methanol and stored at −30°C until staining. Larvae were washed with 0.5% PBT five times. Permeabilization was performed by treating samples with distilled water for 5 min and then with cold acetone (−30°C) for 5 min. Larvae were washed with 0.5% PBT three times and blocked with 5% goat serum in 0.5% PBT for 1 h. Larvae were incubated with primary antibodies diluted in 5% normal goat serum in 0.5% PBT at 4°C overnight. Primary antibodies are rabbit polyclonal anti-Lyz antibody (AnaSpec, AS-55633; 1:200 dilution), mouse monoclonal anti-Cyp7a1 antibody (Merck Millipore, MABD42 clone 15B9.1; 1:200 dilution) and rabbit polyclonal anti-mCherry antibody (GeneTex, GTX128508; 1:200 dilution). After washing with 0.5% PBT, samples were incubated with secondary antibody, Alexa-Fluor-568-conjugated anti-rabbit IgG (Life Technologies; 1:200 dilution) and/or Alexa-Fluor-488-conjugated anti-mouse IgG (Life Technologies; 1:200 dilution) at room temperature for 1 h. Larvae were counterstained with Hoechst 33342 (Life Technologies; 1:2000 dilution) and mounted with PBS containing 80% glycerol. Fluorescent images were obtained with a Nikon A1R confocal laser microscope (Nikon). Cyp7a1 signal intensity in the liver (identified as mCherry-positive area) was quantified from single optical sections using ImageJ software.

### Paraffin sectioning and HE staining

Zebrafish larvae were fixed in 4% PFA in PBS at 4°C overnight. Fixed larvae were dehydrated by a series of diluted ethanol (70, 80, 90, 99.5 and 100%) and xylene. Paraffin filtration was performed at 65°C overnight, and then samples were embedded in paraffin at room temperature. Paraffin sectioning (thickness: 5 µm) was performed with an HM 340E Rotary Microtome (Thermo Fisher Scientific). Sections posterior to the pancreas were collected and deparaffinized by xylene and ethanol treatments, and then stained with Mayer's Hematoxylin and eosinY (Wako Pure Chemical Industries, Osaka, Japan; Wako). Images were taken using a Nikon ECLIPSE Ni-E microscope (Nikon).

### Imaging of neutrophils using *Tg(lyz:EGFP)*

The sibling controls and tumor-bearing larvae carrying *Tg(lyz:EGFP)* and *Tg(fabp10a:mCherry)* or *Tg(fabp10a:mCherry-P2A-cyp7a1)* were obtained from the same clutch. At 7 dpf, larvae were given an anesthetic by 0.008% MS-222 and mounted in 1% NuSieve GTG Agarose (Lonza) in egg water. Fluorescent images of the left side of the liver were obtained using a Nikon A1R confocal laser microscope (Nikon). Liver size was measured using ImageJ software (Schneider et al., 2012). The number of neutrophils overlapping with mCherry signals (i.e. the liver) were manually counted using ImageJ software in all sections containing the liver (6 µm interval).

### Whole-mount *in situ* hybridization

To obtain templates of antisense DIG-labeled RNA probes, coding sequences of interests were amplified using KOD Plus Neo (TOYOBO) from cDNA and then cloned into *Sma*I-digested pBlueScript II SK(−) plasmid, followed by sequence validation (FASMAC). Antisense DIG-labeled RNA probes were synthesized using T3 or T7 RNA polymerase (Roche) and DIG RNA Labeling mix (Roche). At 7 dpf, larvae ware fixed in 4% PFA in PBS at 4°C overnight. Larvae were washed with PBS five times and treated with 3% hydrogen peroxide in 0.5% sodium hydride at room temperature to bleach pigments. After removing pigments, larvae were washed with PBS, and then transferred into methanol and stored at −30°C until staining. Whole-mount *in situ* hybridization was performed as described (Takada et al., 2017) with only a slight modification in that we performed proteinase K treatment by 10 µg/ml concentration at 25°C for 45 min. Images were taken with a Leica DFC310 FX camera.

### Liver size measurement in larvae overexpressing *kras^G12D^* in the liver

*Tg(Liver-Gal4)^Tg/+^; Tg(UAS:RFP)^Tg/+^* and *Tg(5×UAS:EGFP-P2A-kras^G12D^)^+/Tg^* lines were mated to obtain the sibling controls and larvae overexpressing *kras^G12D^* in the liver. At 7 dpf, the RFP signal was imaged with a Leica DFC310 FX camera. Liver size was measured by ImageJ software.

### Body length measurement

*Tg(5×UAS:EGFP-P2A-kras^G12D^)^+/Tg^; Tg(pInt-Gal4)^+/Tg^* and the sibling controls were obtained from the same clutch. Embryos and larvae were reared in a plastic Petri dish in the presence of egg water without foods. At 7 or 9 dpf, zebrafish larvae were given an anesthetic by 0.008% MS-222 and phenotyped into tumor-bearing larvae and the sibling controls. Larvae were placed on the bottom of a plastic Petri dish and lateral view images were taken with a Leica DFC310 FX camera. Lengths of the lateral side views were measured by ImageJ software.

### Oil Red O staining

Oil Red O was purchased from Wako and the experiments were performed essentially as described previously (Kim et al., 2013), except that we did not perform a rinse with 2-propanol after Oil Red O treatment.

### Survival assay

Twenty larvae were reared in a tank from 7 to 14 dpf. Sibling controls and tumor-bearing larvae (Fig. 5I) and tumor-bearing larvae with or without *cyp7a1* overexpression in the liver (Fig. S10B) were used. Larvae were fed the Hikari Lab 130 food (KYORIN). The numbers of live and dead larvae were counted every day. Statistical significance was tested using the log-rank test.

### RNA-seq and bioinformatic analysis

RNA-seq analyses were performed as described previously (Kawaoka et al., 2013; Suzuki et al., 2014). At 7 dpf, larvae were dissected under a microscope. The liver, intestine and the rest of the body from ∼20-30 tumor-bearing larvae or the sibling controls were pooled and RNA extracted. Pooling multiple larvae for preparing sequencing libraries was important to obtain sufficient amount of high-quality RNAs and to minimize the risk to obtain outliers that cannot represent the clutch used. The obtained gene list with reads per million per a kilobase (RPKM) scores are shown in Table S2. To identify differentially expressed genes (DEGs), we first focused on the well-annotated protein-coding genes. RPKM scores were used to calculate the RPKM ratio of tumor/control. In this calculation, 1 was added to all RPKM scores to ignore the scores below ‘1’, and to make analyses more stringent. Recognizing that our dissection cannot prevent cross-contamination, genes showing more than 0.8-fold-enrichment and >0 RPKM in the tissue of interest were further considered. The obtained ratios were used to sort genes to find potential DEGs. As an initial screening to identify reliable DEGs, we focused on a set of genes showing more than 3-fold changes in the RNA-seq experiments. Considering possible differences among clutches, the RNA-seq experiment was followed by qPCR validation with samples prepared from different clutches. Thus, the RNA-seq experiment functioned as a screening to identify DEGs. Data visualization was done mostly using ggplot2 (http://ggplot2.org/). In the main figures, we show genes validated by qPCR. In our experience with our dataset, the validation rate was high for genes with more than 3-fold changes in the intestine-derived samples. In the liver and rest of the body, ‘3-fold criteria’ was not enough to obtain a high validation rate [i.e. genes showing more than 3-fold changes such as *pklr* failed to be validated by qPCR (data not shown)]. The in-house R scripts used in this study are all available upon request. RNA-seq data published in the present study have been deposited under the accession number of DRA005199 in DDBJ (DNA Data Bank of Japan).

### Metabolite measurement

For measuring total BA, single zebrafish larvae were homogenized in 500 µl of chloroform:methanol (1:1) solution to extract total lipids. Samples were centrifuged at 20,000 ***g*** for 20 min at room temperature. Supernatants were collected and evaporated. Dried samples were dissolved in 75 µl of R1 reagent of total Bile Acids Assay Kit (DIAZYME, CA, USA), and then 25 µl of R2 reagent were added. Absorbance at 405 nm was measured by Multiskan GO (Thermo Fisher Scientific). A standard curve was generated using a dilution series of standard bile acids due to unavailability for BA (5-α cyprinol in this case). 3-α hydroxysteroid dehydrogenase equipped in the kit is able to catalyze both bile acids and BAs. Thus, we report obtained scores as relative index (total BA levels measured using bile acids as standards). Note that we have confirmed that *cyp7a1* knockouts abolished total BA levels (Fig. S7G), suggesting that what we measure in this experiment is BA. For Fig. S7G, pellets after centrifugation at 20,000 ***g*** for 20 min at room temperature were further treated with proteinase K to prepare genomic DNA, followed by genotyping.

For cholesterol measurements, single zebrafish larvae were homogenized in 500 µl of chloroform:methanol (2:1) solution to extract total lipids. Samples were centrifuged at 20,000 ***g*** for 20 min at room temperature. Supernatants were collected and evaporated. Dried samples were dissolved in 100 µl of the assay reagent of the Wako cholesterol E-test. Absorbance at 600 nm was measured by Multiskan GO. A standard curve was generated using a dilution series of standard cholesterol. The obtained data were shown as box plots generated using ggplot2 for Figs 6 and 7. Used in-house R scripts are all available upon request.

### Statistics and sample size determination

The values of the bar graphs are expressed as means±s.e.m. The error bars (s.e.m.) shown for all results were derived from biological replicates. Significant differences between two groups were examined using one- or two-tailed unpaired *t*-test. One-tailed test was chosen when we had a hypothesis regarding direction of changes (increased or decreased) in experiments. Statistical significance is assumed if *P*<0.05. The sample size was not pre-determined and was chosen as follows. First, the number of animals was minimized as much as possible in light of animal ethics. Second, ≥80-90% power was favored. Third, in most cases, *n*≥5 was set as a threshold according to the previous reports (Krzywinski and Altman, 2014). For analyzing the growth defect phenotype, with the estimated size effect (around 0.98- to 0.99-fold), a larger sample size (e.g. *n*>50) was preferred to obtain appropriate statistical power. We did not find an apparently abnormal distribution throughout the study except for the controls in Fig. 2S and Fig. S2J, where the majority of controls exhibit 0, and Fig. S5E. No data exclusion was performed. No blinding was performed.

## Supplementary Material

Supplementary information
