# Supplementary material for: A novel zebrafish intestinal tumor model reveals a role for cyp7a1-dependent tumor–liver crosstalk in causing adverse effects on the host
Source: Dis Model Mech. 2018 May 3;11(8):dmm032383. doi: 10.1242/dmm.032383 (PMC6124559; doi:10.1242/dmm.032383)
Supplement: Supplementary information [file dmm-11-032383-s1.pdf]

Enya et al. Fig. S1

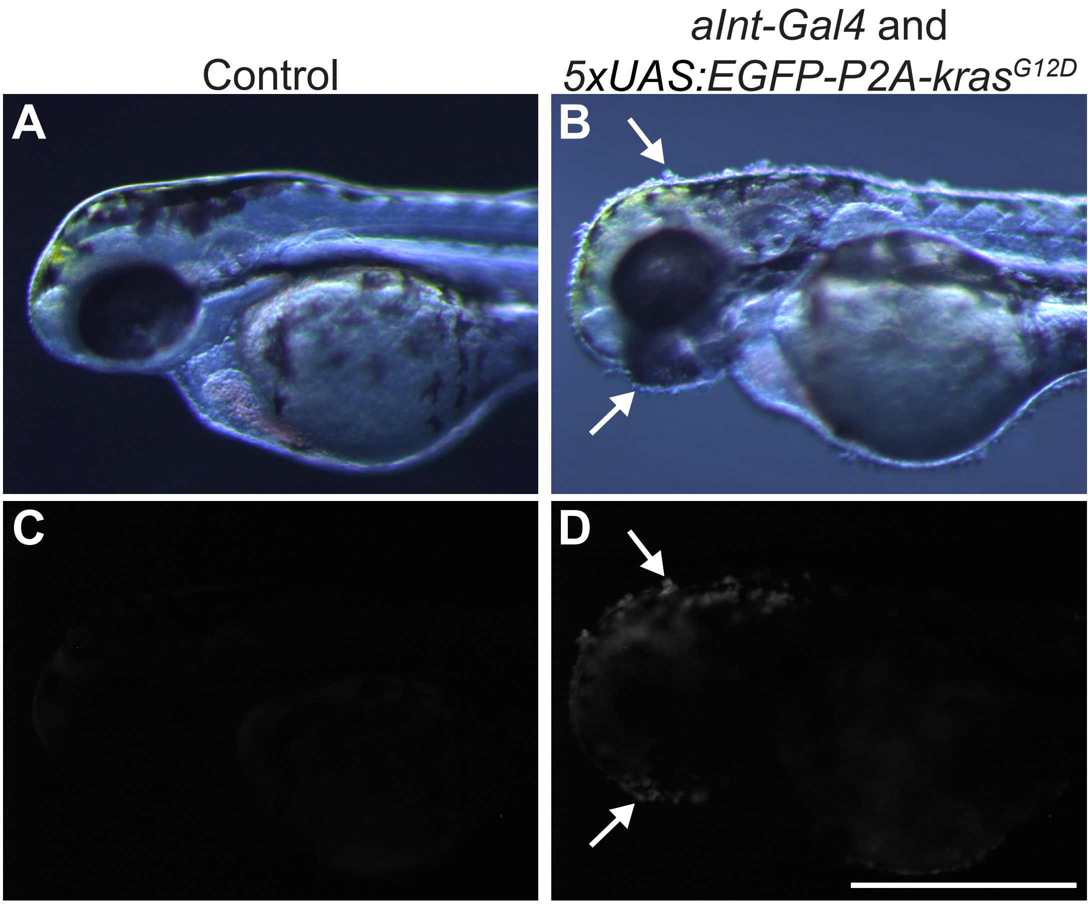

Figure S1 *aInt-Gal4* is expressed in the epidermis at 2 dpf

Bright filed (A, B) and EGFP (C, D) images of the sibling controls and *EGFP-P2A-kras*^{G12D}-expressing larvae driven by gSAIzGFFM103B (*aInt-Gal4*) at 2 dpf. White arrows indicate *EGFP-P2A-kras*^{G12D}-expressing cells. Scale bar represents 500 µm.

Enya et al. Fig. S2

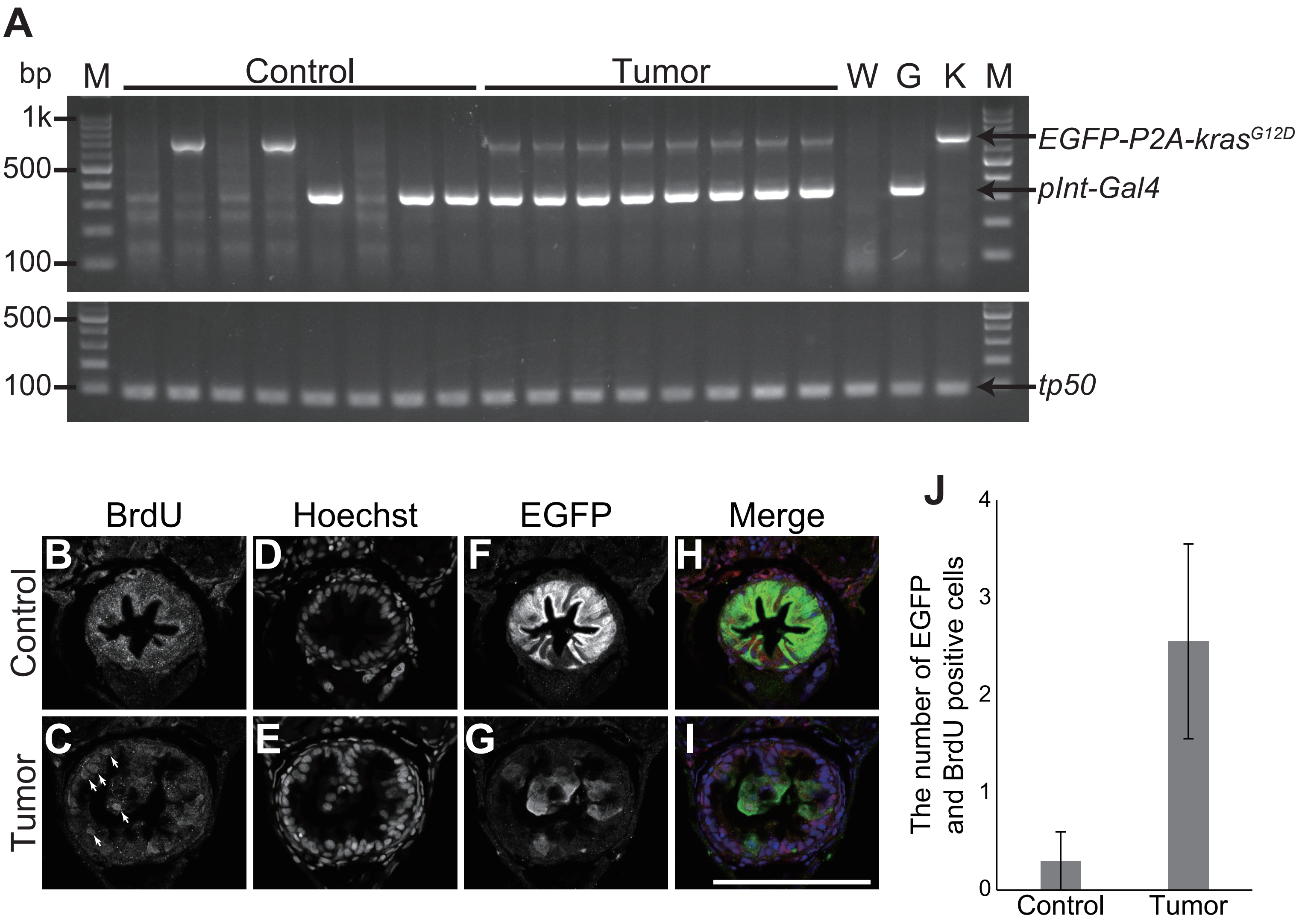

Figure S2 Characterization of the pInt-Gal4-driven tumor model

(A) A gel image of genotyping of tumor-bearing larvae. Band sizes detecting *Tg(5×UAS:EGFP-P2A-kras^{G12D})*, *Tg(pInt-Gal4)* and *tp53* are 701 bp, 345 bp and 88 bp, respectively. *tp53* locus is used as a PCR control. M, DNA ladder marker: W, wild type larvae: G, parental *Tg(pInt-Gal4)* line: K, parental *Tg(5×UAS:EGFP-P2A-kras^{G12D})* line.

(B)-(I) Representative images of fluorescent immunohistochemistry for BrdU and EGFP in transversal sections of the posterior intestine of the sibling controls and tumor bearing larvae at 5 dpf. BrdU (B, C), Hoechst33342 (D, E) and EGFP (F, G) images are shown. In the merged images (H, I), BrdU, Hoechst33342 and EGFP signals are shown in red, blue and green, respectively. White arrows indicate intestinal cells positive for BrdU, Hoechst33342, and EGFP. Scale bar indicates 100 μm.

(J) The number of BrdU and EGFP positive intestinal cells. The number of BrdU and EGFP positive cells was counted from single section per individual larva. The data harbors 10 and 9 biological replicates from the sibling controls and tumor-bearing larvae, respectively. Error bars represent ± s.e.m.

Disease Models & Mechanisms • Supplementary information

Enya et al. Fig. S3

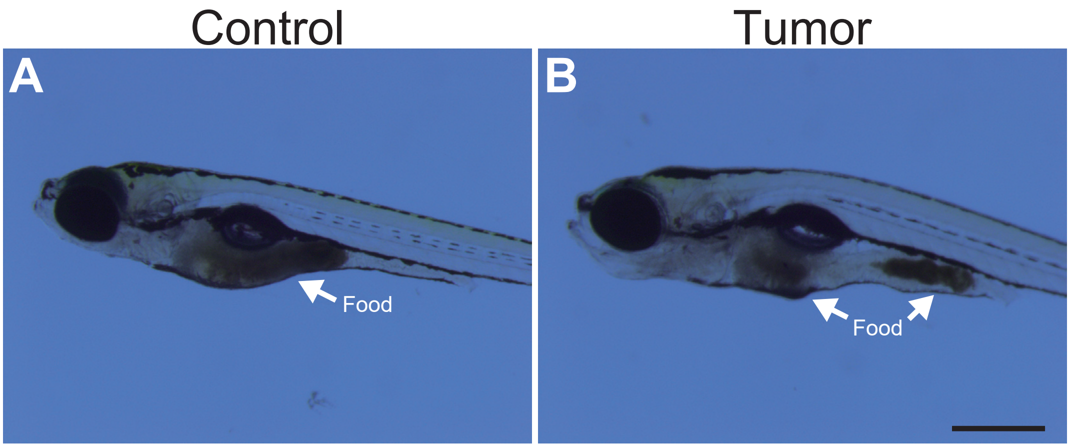

Figure S3 The intestinal rumen is not completely disrupted in tumor-bearing larvae

Representative images of the sibling controls (A) and tumor-bearing larvae (B) at 9 dpf in the presence of foods in the intestine are shown. Scale bar represents 500 μm.

Disease Models & Mechanisms • Supplementary information

Enya et al. Fig. S4

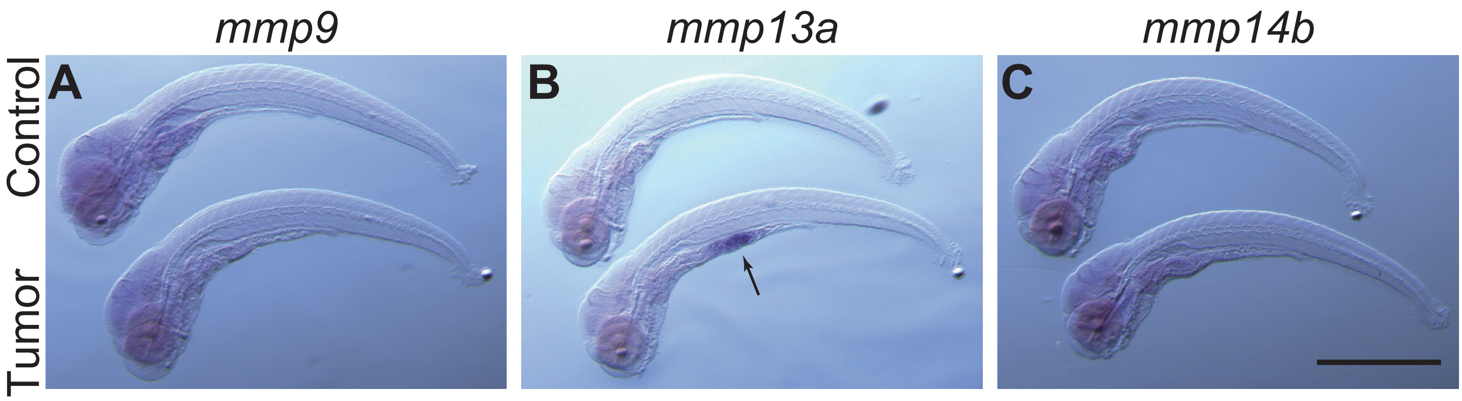

Figure S4 Whole mount *in situ* hybridization experiments for *mmp* genes

qPCR results shown in Fig. 3M-3O were validated with the aid of *in situ* hybridization experiments for (A) *mmp9*, (B) *mmp13a*, and (C) *mmp14b* at 7 dpf. Increased expression of *mmp13a* (indicated by an arrow), the most highly expressed gene among these three, was confirmed. We could not detect expression of the other two genes in our hands probably due to lower expression levels. Shown are the representative images chosen from approximately 10 larvae. Scale bar indicates 500 µm.

Enya et al. Fig. S5

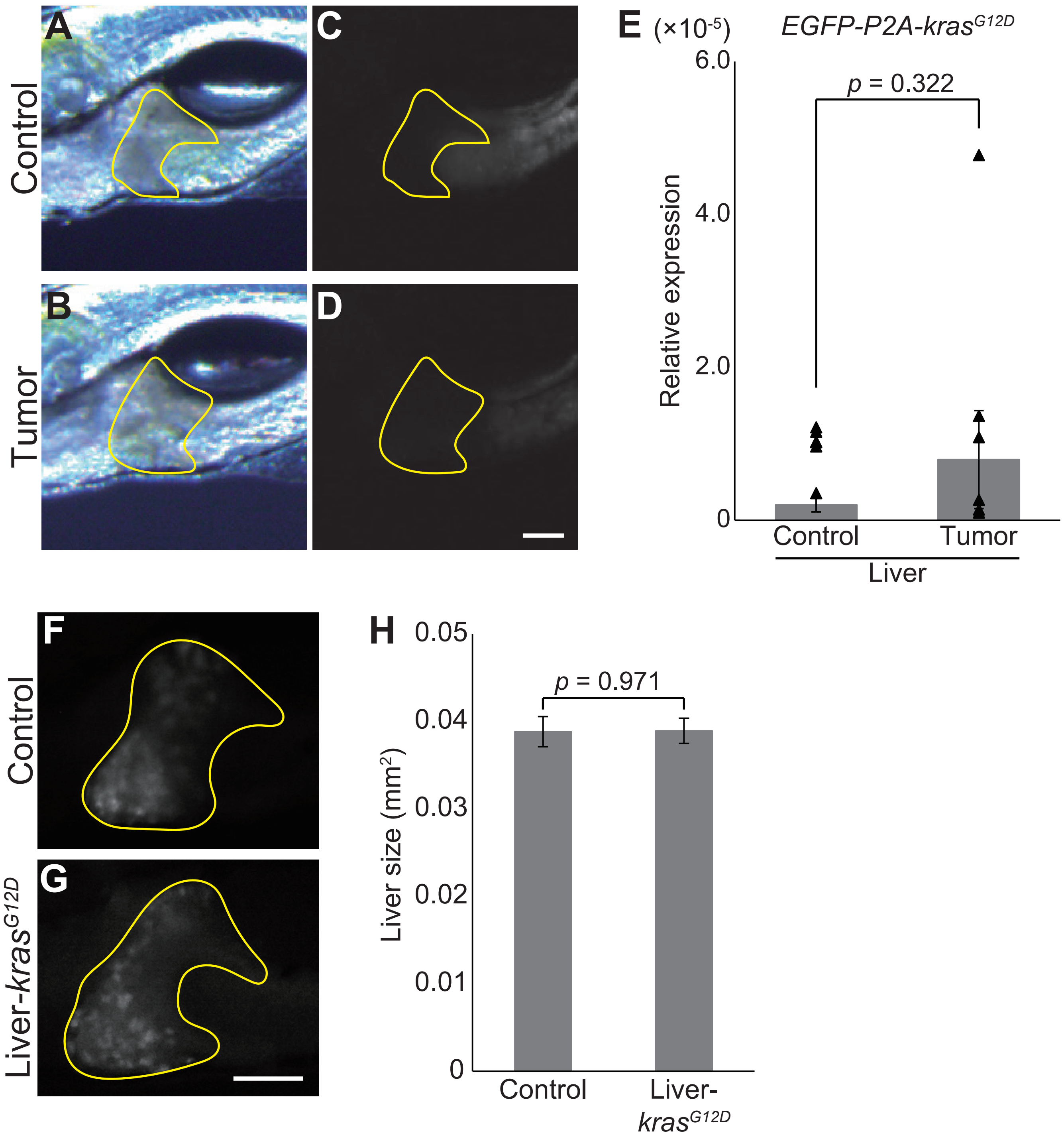

Figure S5 Hepatomegaly phenotype is not caused by leaky expression of *kras*^{G12D} in the liver (A)-(D) Representative images for the livers of sibling controls and tumor-bearing larvae. Bright field images (A-B) and EGFP images (C-D) are shown. The liver is encircled by yellow lines. Scale bar indicates 100 μm.

(E) qPCR analysis for *EGFP-P2A-kras*^{G12D} in the liver. Because the transgene lacked intron, total RNAs were DNase-treated. In addition, samples without reverse transcription were included. The scores are normalized to expression of *rpl13a*. The data harbors 6 biological replicates, each containing 5 larvae. Bars and dots represent averages and each value, respectively. Error bars represent ± s.e.m. Statistical significance was tested using student's *t*-test (unpaired, two-tailed). Although not excluded, note that there is a potential outlier in data from tumor-bearing larvae.

(F)-(G) Representative RFP images for the livers of the sibling controls (F) and larvae expressing *kras*^{G12D} driven by Liver-Gal4 (G) at 7 dpf. The liver is encircled by yellow lines. Scale bar indicates 100 μm.

(H) Liver size of the sibling controls and *kras*^{G12D}-expressing larvae at 7 dpf. The liver was identified by the RFP signal and liver size was measured using ImageJ software. The data harbors 12 biological replicates. Error bars represent ± s.e.m. Statistical significance was tested using student's *t*-test (unpaired, two-tailed).

In (A)-(E), sibling controls (Control) represent *Tg(pInt-Gal4)*$^{+/Tg}$; *Tg(UAS:EGFP)*$^{+/Tg}$ and tumor-bearing larvae (Tumor) represent *Tg(pInt-Gal4)*$^{+/Tg}$; *Tg(5×UAS:EGFP-P2A-kras*^{G12D}*)*$^{+/Tg}$. In (F)-(G), sibling controls (Control) represent *Tg(Liver-Gal4)*$^{+/Tg}$; *Tg(UAS:RFP)*$^{+/Tg}$ and Liver-*kras*^{G12D} represents *Tg(Liver-Gal4)*$^{+/Tg}$; *Tg(UAS:RFP)*$^{+/Tg}$; *Tg(5×UAS:EGFP-P2A-kras*^{G12D}*)*$^{+/Tg}$.

Disease Models & Mechanisms • Supplementary information

Enya et al. Fig. S6

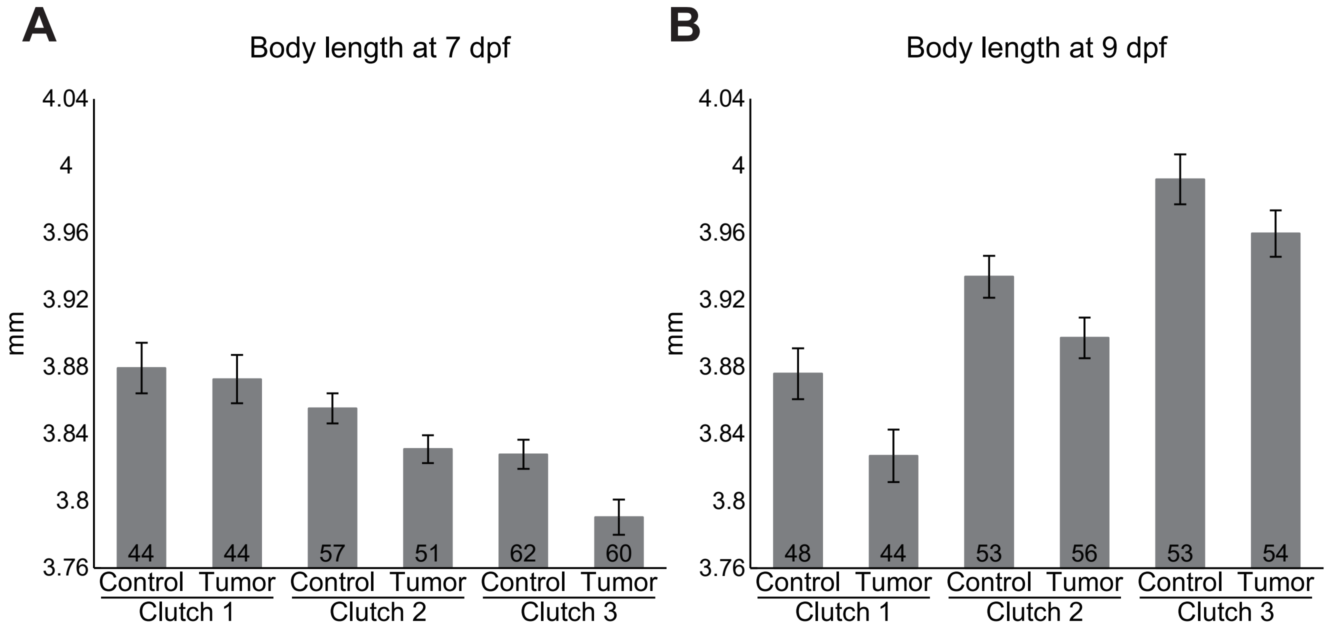

Figure S6 The intestinal tumor causes systemic growth defects in multiple independent clutches

Body length data from three individual clutches at 7 dpf (A) and 9 dpf (B) are presented. Error bars represent ± s.e.m. The numbers within the bars indicate the number of biological replicates in each clutch.

Enya et al. Fig. S7

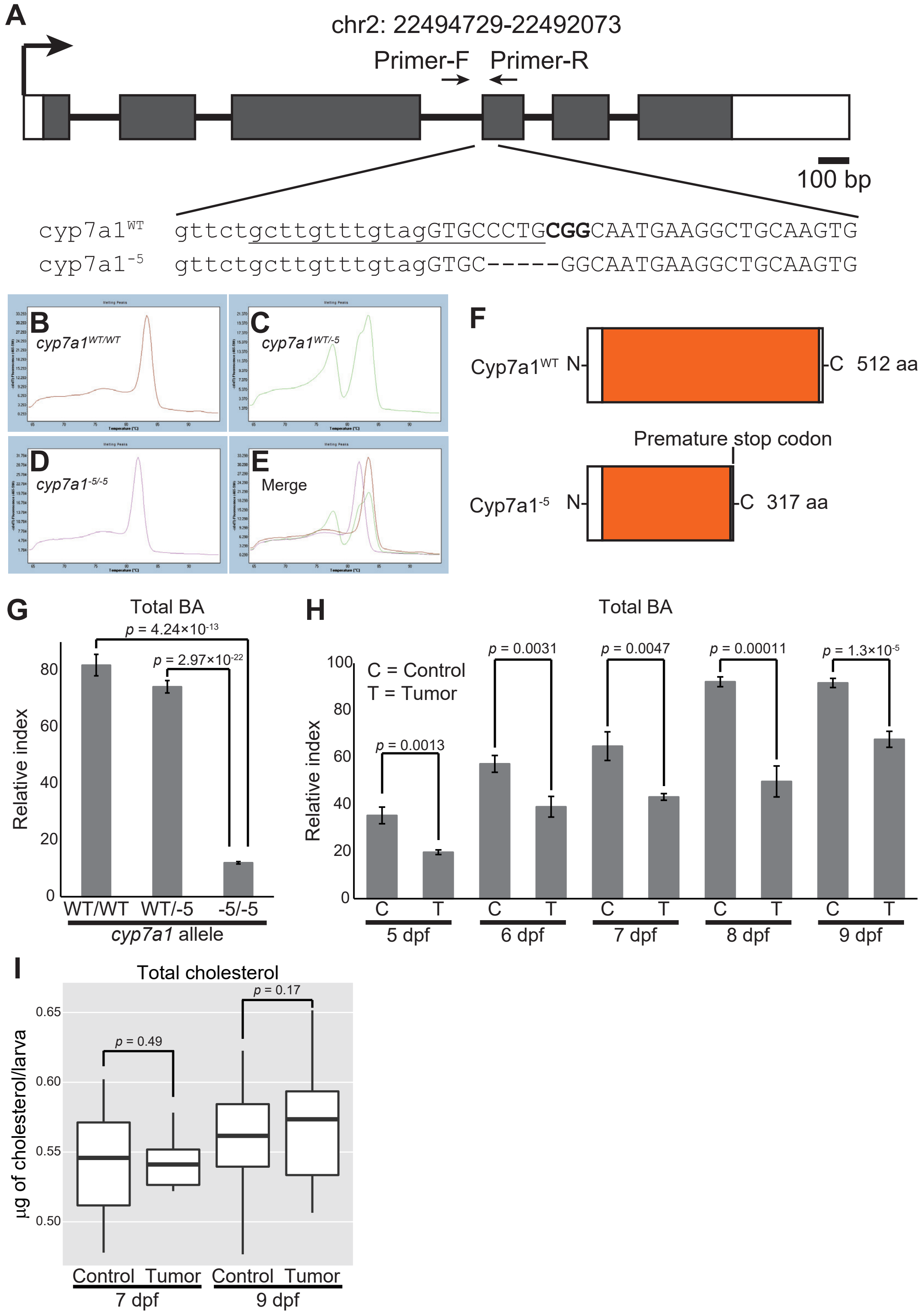

Figure S7 *cyp7a1* is critical for total BA synthesis in zebrafish

(A) The gene structure for zebrafish *cyp7a1*. White and gray boxes and lines indicate untranslated regions, coding sequence and intron respectively. The primers used for genotyping are shown. The sequence of newly established *cyp7a1*$^{-5}$ allele, which lacks 5 bp in 4th exon, is also shown. Sequences of intron and exon are written in lower and upper cases, respectively. Underlined and bold characters indicate target for CRISPR-Cas9 and PAM sequences, respectively.

(B)-(E) Representative results of high resolution melting (HRM) assay for genotyping the *cyp7a1* allele.

(F) The structure of Cyp7a1^{WT} and Cyp7a1^{-5} proteins. Orange box indicates Cyotochrome P450 domain searched by Pfam (http://pfam.xfam.org/). Predicted Cyp7a1^{-5} protein lacks 201 aa due to premature stop codon. Extra 6 aa at C-terminus are shown in gray box.

(G) Measurement for systemic bile alcohol levels of *cyp7a1* mutants at 7 dpf (n = 18 for WT/WT, 27 for WT/-5, and 15 for -5/-5). The scores are relative index determined using bile acids as standards (see materials and methods). Statistical significance was tested using student's *t*-test (unpaired, one-tailed). Note that *cyp7a1*$^{-5/-5}$ homozygous mutant did not show any obvious developmental and morphological defects at least at 0-7 dpf.

(H) Total bile alcohol levels of the sibling controls and tumor-bearing larvae at 5-9 dpf (n = 8 for each). Statistical significance was tested using student's *t*-test (unpaired, one-tailed).

(I) Measurement for systemic cholesterol levels at 7 and 9 dpf (n = 12 for 7 dpf and 16-19 for 9 dpf). Statistical significance was tested using student's *t*-test (unpaired, one-tailed).

Disease Models & Mechanisms • Supplementary information

Enya et al. Fig. S8

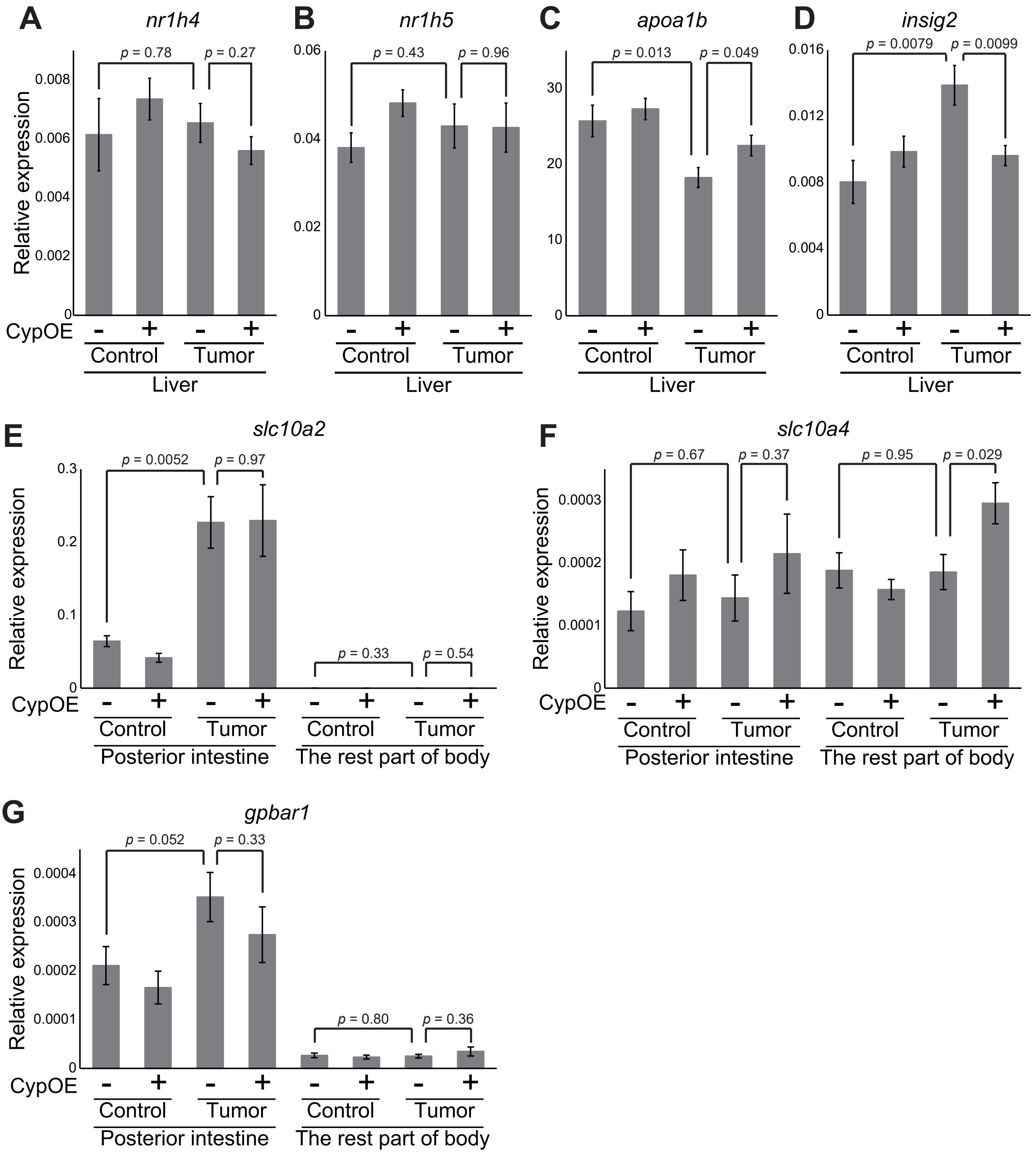

Disease Models & Mechanisms • Supplementary information

Figure S8 Gene expression analyses for bile-related genes

(A)-(D) qPCR analyses for Farnesoid X receptors (FXRs; *nr1h4* and *nr1h5*) (A-B), and known FXR targets (*insig2* and *apoa1b*) (C-D) in the liver at 7 dpf. The data harbors 6 biological replicates, each containing 5 larvae. Error bars represent ± s.e.m. Statistical significance was tested using student's *t*-test (unpaired, two-tailed). Note that expression of *insig2* inversely correlates with total BA levels. CypOE - and + indicate the absence and presence of *Tg(fabp10a:mCherry-P2A-cyp7a1)*, respectively.

(E)-(G) qPCR analyses for bile transporters (*slc10a2* and *slc10a4*) (E-F), the G protein-coupled bile acid receptor (*gpbar1*) (G) in the posterior intestine and the rest part of body at 7 dpf. The data harbors 5-6 biological replicates, each containing 3 larvae. Error bars represent ± s.e.m. Statistical significance was tested using student's *t*-test (unpaired, two-tailed). Note that expression of *slc10a2* in the intestine is strongly affected by expression of $kras^{G12D}$. CypOE - and + indicate the absence and presence of *Tg(fabp10a:mCherry-P2A-cyp7a1)*, respectively.

Disease Models & Mechanisms • Supplementary information

Enya et al. Fig. S9

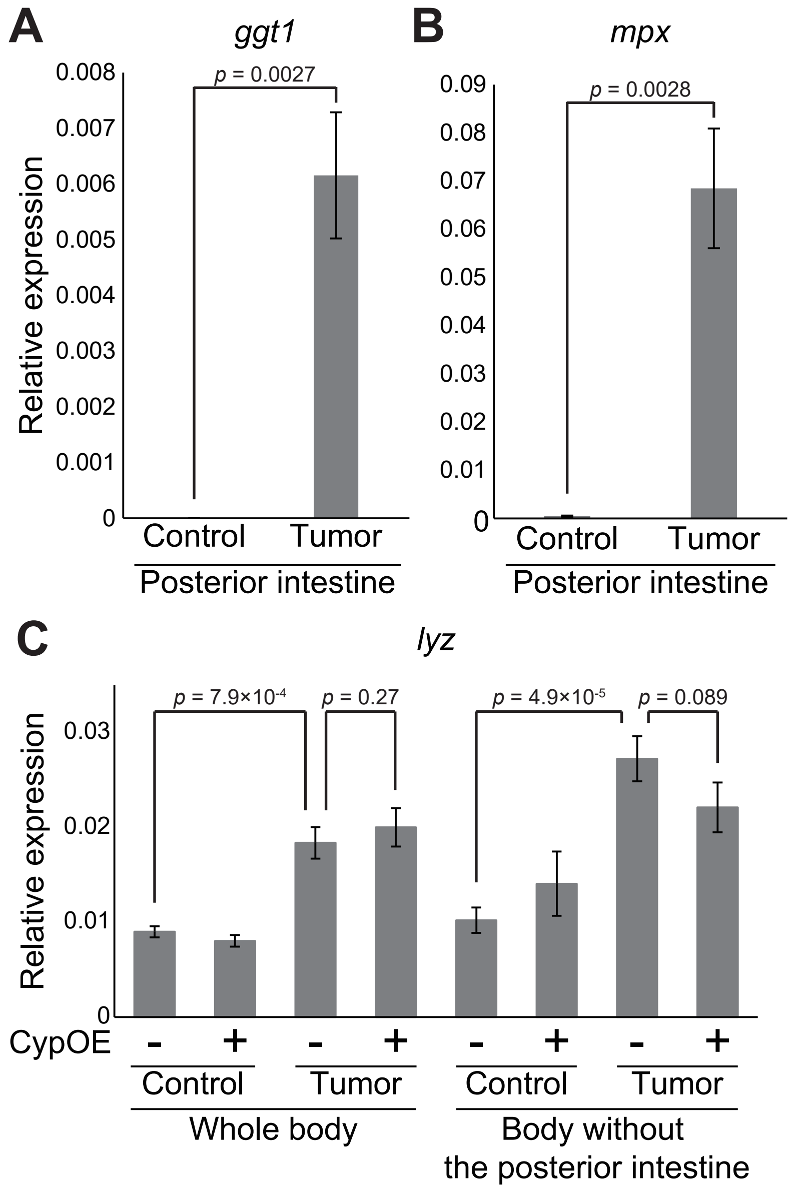

Figure S9 Gene expression analyses for *ggt1*, *mpx*, and *lyz*.

(A)-(B) Gene expression levels of *ggt1* (A) and *mpx* (B) in the intestine at 9 dpf are shown. The scores are normalized by expression of *rpl13a*. The data harbors 5 biological replicates, each containing 5 larvae. Error bars represent ± s.e.m. Statistical significance was tested using student's *t*-test (unpaired, one-tailed).

(C) qPCR analyses for *lyz* in the whole body and body without the posterior intestine at 7 dpf. The data harbors 6 biological replicates, each containing 3-5 larvae. Error bars represent ± s.e.m. Statistical significance was tested using student's *t*-test (unpaired, one-tailed).

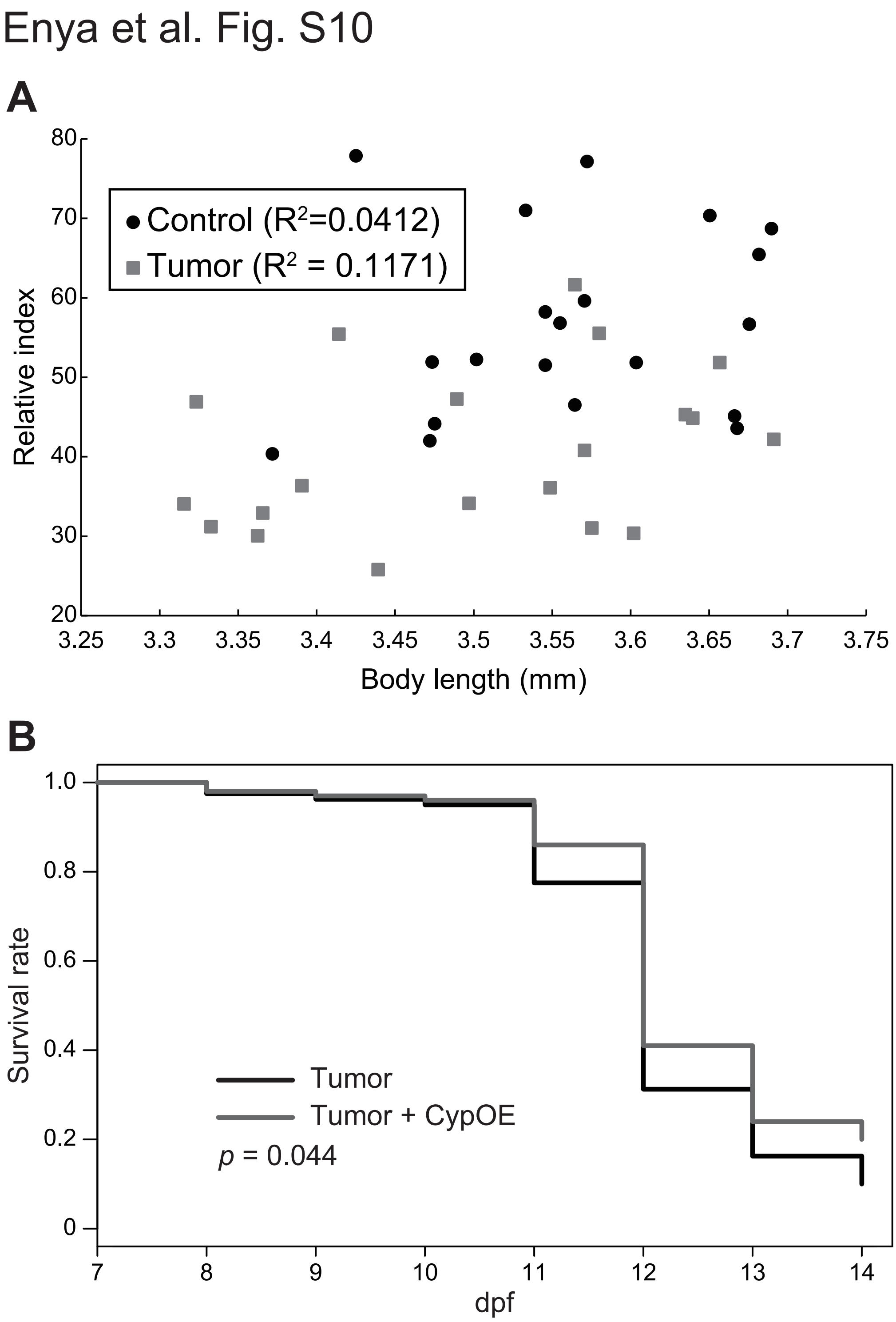

Figure S10 Correlation between body length and total BA levels and effect of *cyp7a1* overexpression in the liver on zebrafish survival

(A) Correlation between body length and total bile alcohol levels at 9 dpf (n = 20 per a group).

Disease Models & Mechanisms • Supplementary information

(B) Survival rates of tumor-bearing larvae with or without *cyp7a1* overexpression in the liver from 7 to 14 dpf are shown by the Kaplan-Meier curve. Data were obtained by three independent experiments. The total numbers of analyzed larvae are 80 (without *cyp7a1* over expression) and 100 (with *cyp7a1* overexpression). Overexpression of *cyp7a1* in the liver only had marginal effect on zebrafish survival, and results varied among clutches (data not shown). Statistical significance was tested using the log rank test. + CypOE indicates the presence of *Tg(fabp10a:mCherry-P2A-cyp7a1)*.

Disease Models & Mechanisms • Supplementary information

Table S1 The primers and DNA sequences used in the study

Click here to Download Table S1

Table S2 Raw RPKM scores determined by RNA-seq analysis

Click here to Download Table S2

Table S3 Calculation for sample enrichments

Click here to Download Table S3

Table S4 The list of 8261 liver enriched genes (Liver to body > 0.8, Control-liver > 0)

Click here to Download Table S4

Table S5 The list of 7294 body-enriched genes (Body to liver > 0.8, Body to intestine > 0.8m Control-body > 0)

Click here to Download Table S5

Table S6 The list of 8002 intestine-enriched genes (Intestine to body > 0.8, Control-intestine > 0)

Click here to Download Table S6

Disease Models & Mechanisms • Supplementary information
